# Companion diagnostic requirements for spatial biology using multiplex immunofluorescence and multispectral imaging

**DOI:** 10.3389/fmolb.2023.1051491

**Published:** 2023-02-09

**Authors:** Darren Locke, Clifford C. Hoyt

**Affiliations:** ^1^ Clinical Assay Development, Akoya Biosciences, Marlborough, MA, United States; ^2^ Translational and Scientific Affairs, Akoya Biosciences, Marlborough, MA, United States

**Keywords:** predictive biomarker, immuno-oncology, cell phenotyping, phenoptics, multiplex immunofluorescence, clinical workflow, image analysis, spatial biology

## Abstract

Immunohistochemistry has long been held as the gold standard for understanding the expression patterns of therapeutically relevant proteins to identify prognostic and predictive biomarkers. Patient selection for targeted therapy in oncology has successfully relied upon standard microscopy-based methodologies, such as single-marker brightfield chromogenic immunohistochemistry. As promising as these results are, the analysis of one protein, with few exceptions, no longer provides enough information to draw effective conclusions about the probability of treatment response. More multifaceted scientific queries have driven the development of high-throughput and high-order technologies to interrogate biomarker expression patterns and spatial interactions between cell phenotypes in the tumor microenvironment. Such multi-parameter data analysis has been historically reserved for technologies that lack the spatial context that is provided by immunohistochemistry. Over the past decade, technical developments in multiplex fluorescence immunohistochemistry and discoveries made with improving image data analysis platforms have highlighted the importance of spatial relationships between certain biomarkers in understanding a patient’s likelihood to respond to, typically, immune checkpoint inhibitors. At the same time, personalized medicine has instigated changes in both clinical trial design and its conduct in a push to make drug development and cancer treatment more efficient, precise, and economical. Precision medicine in immuno-oncology is being steered by data-driven approaches to gain insight into the tumor and its dynamic interaction with the immune system. This is particularly necessary given the rapid growth in the number of trials involving more than one immune checkpoint drug, and/or using those in combination with conventional cancer treatments. As multiplex methods, like immunofluorescence, push the boundaries of immunohistochemistry, it becomes critical to understand the foundation of this technology and how it can be deployed for use as a regulated test to identify the prospect of response from mono- and combination therapies. To that end, this work will focus on: 1) the scientific, clinical, and economic requirements for developing clinical multiplex immunofluorescence assays; 2) the attributes of the Akoya Phenoptics workflow to support predictive tests, including design principles, verification, and validation needs; 3) regulatory, safety and quality considerations; 4) application of multiplex immunohistochemistry through lab-developed-tests and regulated *in vitro* diagnostic devices.

## 1 Introduction

**FIGURE 1:**
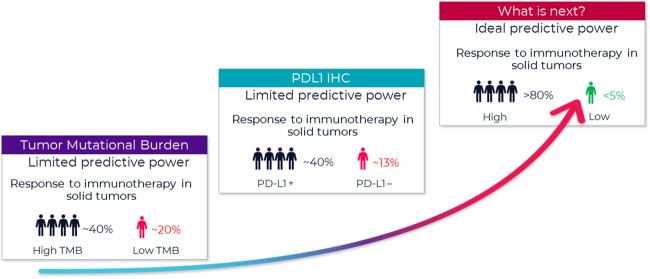
**Growing need to improve prediction of patient response**. FDA approved companion diagnostics show limited predictive value. A new type of biomarker with better predictive power is urgently needed. A biomarker with an ideal predictive power (>80% accuracy) remains a critical missing link to identifying appropriate candidates for immunotherapy and tailoring immunotherapy treatment regimens. One of the new promising biomarkers is tumor mutational burden (TMB) (Hendriks et al., 2018), and those tumors with high TMB may respond best to ICIs. Several studies using samples of patients included in clinical trials as well as retrospective series reported ICI outcome for patients in relation to TMB. That said, outcome on ICI can be influenced by several factors. For example, several tumor and patient characteristics appear to influence response to PD-1/PD-L1 inhibitors, and this must be considered when selecting patients for this treatment (Diggs and Hsueh, 2017). With multiple possible treatment options, biomarkers are needed to identify which subgroup of patients is likely to benefit the most from a certain therapy.

In immuno-oncology (IO), there is a need for improved biomarkers to predict who will respond to treatment. While immunotherapy may lead to complete remission in some patients, average response rates continue to remain within the 20%–30% range^1^ (Figure 1). An emerging new biomarker class in the tumor microenvironment (TME) are Spatial Phenotypic Signatures (SPS), which are defined by the measurement of the cell densities and interactions between tumor and immune cells using multiplex immunohistochemistry (mIHC) (Hoyt, 2021).

Multiplexing resolves clinical problems insufficiently addressed in current diagnostic testing by leveraging multiple biomarkers simultaneously. Tissue image analysis (Parra, 2021) using mIHC highlights the importance of spatial biology, in demonstrating cell relationships and identifying SPS between certain biomarkers, for understanding a patient’s likelihood to respond to, typically, immune checkpoint inhibitor (ICI) therapies. Context matters.

The limitations of chromogenic IHC methods have made the implementation of mIHC in clinical trial settings challenging, particularly when incorporating more than three biomarkers. When multiple markers are co-localized, spectral absorption characteristics of brightfield (BF) dyes typically prevent reliable unmixing for per-target quantitation (van der Loos, 2008). This is a problem less seen when using fluorescence (FL)-based methods, although overlapping spectral signatures can muddy the information captured at each wavelength within each pixel of an image.

**FIGURE 2:**
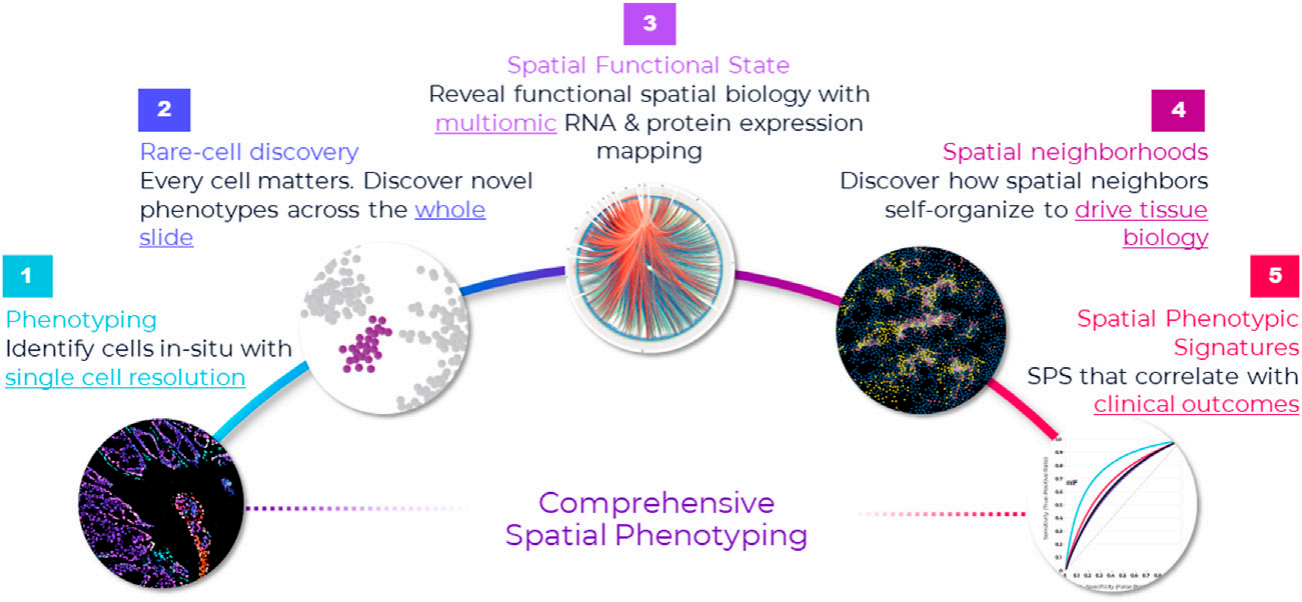
**Comprehensive framework for spatial applications**. Spatial applications depend on the type of study that the researcher is engaged in. This comprehensive framework captures the continuum of needs across discovery, translational and clinical research. Starting from the far left, the first step in spatial biology is phenotyping cells *in situ*. In many ways, this is a foundational element in any spatial biology study—map cells with spatial context. This is the starting point, and it requires single-cell resolution. If the goal is to discover novel cell types or rare cells, then it warrants an unbiased approach to discovery—that is, mapping every single cell in the tissue through whole slide imaging. This approach not only provides a macro-level view of the tissue architecture but also a micro-level view into each cell and is necessary for uncovering extremely rare cell types—down to less than <0.1% abundance (e.g., Nguyen et al., 2018). Once cells are phenotyped, they can be mapped to distinct tissue substructures, called cellular neighborhoods, based on spatial interactions and how the cells cluster. Cellular neighborhoods are emerging as a seminal concept in spatial analysis because of their correlation to cancer progression and treatment response (e.g., Schurch et al., 2020). On the translational/clinical side of the spectrum, far right, the goal is discovering spatial biomarker signatures (e.g., Theobald et al., 2018; Badve et al., 2021; Griffin et al., 2021) and establishing their clinical significance, which requires studying large cohorts and a high throughput approach.

Prevailing higher-plex discovery platforms in cancer research that use different detection modalities can multiplex 10 s of proteins iteratively or simultaneously. However, test throughput and economics are not well suited to translational workflows and unlikely to change despite approaches being technically compelling. Instead, these methods will provide a rich pipeline of new biomarker signatures that can be converted to simpler lower-plex assay panels that are more suitable for clinical trials and translation into eventual standard-of-care (Figure 2).

Several technical approaches exist; details are beyond the scope of this Review (though see Table 1) and are discussed elsewhere (Hofman et al., 2019; McNamara et al., 2020; Tan et al., 2020; Taube et al., 2020; McGinnis et al., 2021; Moffitt et al., 2022).

**TABLE 1:** Multiplex technology providers and topline methods for spatial biology. Multiplex tissue imaging providers, and their use of brightfield-, fluorescence- or DNA- and mass cytometry-based methods for spatial biology.

Technology provider	Top-level summary				
Agilent^a^	RUO, Clinical/IVD solutions provider for IHC, sequential brightfield multiplexing				
Akoya Biosciences^b^	Single-cell, sub-cellular RNA and protein detection using tyramide signal amplification with multispectral whole-slide fluorescence imaging				
10X Genomics^c^	Spot-based transcriptomics using release-ligated probe pairs, with *in situ* protein and multiomics on roadmap				
Cell IDx^d^	Single-cell, sub-cellular protein detection using proprietary anti-hapten antibodies for sequential brightfield and fluorescent multiplexing				
IonPath^e^	Mass spectrometry cytometry with ROI spatial tissue analysis using metal-conjugated antibodies				
Leica^f^	RUO, Clinical/IVD solutions provider for IHC, brightfield and fluorescent multiplexing through partnerships, higher plex fluorescent cyclic multiplexing using dye inactivation methodology				
Lunaphore^g^	High-plex staining platform with proprietary fluidics technology, no reagent solution				
Miltenyi^h^	Flow-centric, moving into spatial proteomics using recombinantly engineered antibody fragments coupled to releasable fluorochromes for sequential fluorescence multiplexing				
Nanostring^i^	Spot-based protein and RNA profiling, using photocleavable oligonucleotide probes, single cell analysis and subcellular resolution on roadmap				
Standard BioTools^j^	Imaging mass cytometry with ROI spatial tissue analysis using CyTOF technology with using metal-conjugated antibodies				
Ultivue^k^	Single-cell, sub-cellular protein detection using DNA barcoded antibodies for sequential fluorescence multiplexing				
Ventana^l^	RUO, Clinical/IVD solutions provider for IHC, predominantly brightfield multiplexing but tyramide/hapten-based signal amplification for fluorescence multiplexing				
Markers	2–3	4–5	6–7	8–9	10+

Intended Use^m^	Translational Diagnostic	Translational Diagnostic	DiscoveryHypothesis Translational	Discovery Hypothesis	DiscoveryHypothesis
Tech Method	Brightfield (absorbance)	Fluorescence (emission)			Barcode/Ion (mass or ID tag)
Example Providers	Agilent, Leica, Ventana	Akoya, Cell IDX, Ultivue, Leica, Ventana			10X Genomics, IonPath, Leica (FL), Miltenyi, Nanostring, Standard BioTools
Phenotypes	Low	Medium	Medium	High	Very High
Dynamic Range	Low—1 Log	Medium—3 Log	Medium—3 Log	Medium—3 Log	High—5 Log
Resolution	200nm, WSI	200nm, WSI	200nm, WSI	200nm, WSI/ROI	1µM, ROI
Throughput	High—0.5 min/mm^2^	Medium—1 min/mm^2^	High—1 min/mm^2^	Low—10 min/mm^2^	Very Low—1 h/mm^2^
Build Complexity	Low	Medium	Medium	High	Very High
Providers^n^	Many	Growing	Growing	Fewer	Few

Assumptions and Notes

^a^ https://www.agilent.com/en/product/immunohistochemistry

^b^ https://www.akoyabio.com/

^c^ https://www.10xgenomics.com/

^d^ https://cellidx.com/

^e^ https://www.ionpath.com/

^f^ https://www.leicabiosystems.com/us/ihc-ish/

^g^ https://lunaphore.com/

^h^ https://www.miltenyibiotec.com/US-en/products/macs-imaging-and-microscopy.html

^i^ https://nanostring.com/

^j^ https://www.standardbio.com/products-services/technologies/imaging-mass-cytometry

^k^ https://ultivue.com/

^l^ https://diagnostics.roche.com/us/en/products/product-category/anatomical-pathology.html

^m^ For definition, see Table 2: Intended use performance requirements for spatial biology using mIF and MSI.

^n^ Includes contract research organizations (CRO) offering technology, as additional solution providers.

Published academic studies routinely demonstrate that lower-plex (4–9 marker) multiplex immunofluorescence (mIF) assays might be sufficiently sensitive, practical, and affordable to see wider-spread clinical adoption. Unfortunately, research-use-only (RUO) application of mIF in translational research lacks the rigorous controls and standardization needed to support the stringent reproducibility, sensitivity, and specificity requirements of those same mIF assays for clinical trial use. A foundation of scientific discovery, rigor is particularly critical when the performance attributes undergird drug discovery or patient therapeutic choice.

Integrating image analysis (IA) solutions with mIHC/mIF assay optimization and validation has seen limited regulatory guidance and fewer harmonization effects. Certain analytic limitations of a wet-lab assay can be ameliorated with IA, but algorithms are susceptible to generating erroneous data (Aeffner et al., 2019). For example, accurately identifying cell subpopulations based on marker co-expression can be confounded by staining variability and artifacts, inherent biological heterogeneity within samples, and among patients, and image variability (such as areas out of focus, incomplete whole slide scans, section thickness, etc) arising from different instrumental (e.g., scanner) approaches for data capture. Reliable classification of cell types and their functional state based on lineage and expression markers is a cornerstone of accurately characterizing immuno-biological activity in the TME.

Progress to date with the application of IA, including artificial intelligence (AI) and machine/deep learning (ML) approaches, has focused mainly on BF IHC and histopathological staining (i.e., hematoxylin and eosin [H&E]—van der Laak et al., 2021), which does not leverage the wealth of data that can be captured through mIHC and provides no information on immune cell subsets within the TME.

“Virtual multiplexing,” which involves digital image alignment and fusion of consecutive serial sections of single-stained or low-multiplex BF assays (multiplex BF; mBF), provides an alternate approach to single-section mIHC. However, analysis of individual protein markers on serial sections is not always supportive of reliable cell type classification based on co-expression and these workflows are often not amenable to high-throughput needs of clinical studies or trials, with a few exceptions (e.g., Hoiberg, 2009).

Formalin-fixed paraffin-embedded (FFPE) tissue is the most for use method for preparing and preserving specimens for use in research and therapeutic development. Data demonstrate that upfront handling and processing of FFPE specimens have a significant impact on the quality of intercellular and intracellular components to affect results of downstream assay and analyses. For IF, it is autofluorescence (AF)—endogenously found in most tissues and enhanced by formalin fixation (e.g., Robertson and Isacke, 2011; Baharlou et al., 2021)—that contributes deleterious noise that can compromise examination of single component IF images, particularly for low abundance targets. FFPE or IHC sample preparation techniques intended to reduce AF prove only partially successful, whereas image-based spectral unmixing technologies can achieve near complete reduction in favorable circumstances (Mansfield et al., 2008).

For any spatially resolved mIF technology to be successful and provide clinically meaningful advantages over other simpler IHC approaches, awareness of these potential stumbling blocks and others placed by pre-analytical variables is important, especially as a greater number of tested biomarkers inform the test readout. Multiplex IHC, in general, also requires greater vigilance in terms of quality control of the many analyte-specific reagents (ASR) that comprise the final assay. The difficulty of finding per-target detection controls that should be incorporated into routine testing practices can reduce the likelihood of reporting errors.

Automated IHC slide staining technologies coupled with visual assessment by pathologists have been readily implemented in routine clinical care settings and have historically delivered high-value medical and clinical information at a relatively low cost. However, useability issues posed by high complexity test interpretation, using intricate analytical and bioinformatical software tools, must be addressed, once they are configured and “locked-down,” particularly by platform providers. Clinical software needs to be developed from the ground up under a robust quality assurance (QA) system and reduced to essential functionality. Choice of code base, architecture, workflow, and user interface are driven by technical and operational requirements of the test. Multiplexed data acquisition pipelines must be streamlined and operationalized to ensure the intended use of multiplex technology to enable rational clinical decisions quickly and accurately with economic benefit.

Regardless, benchmark efforts are underway, steered by national societies and working groups comprised of representatives from academia and biopharma^2^ ^,^ ^3^. Their goal is to develop and support pilot projects that use mIF and other emerging technology platforms with the potential to overcome limitations of established IHC methodologies in the application of multi-dimensional biomarkers.

A new Frontier of biomarker discovery based on spatial biology presents a path toward the clinic, as workflows become practical and analytically robust. Documented design principles that include validation and verification processes will ensure meaningful translation of spatially resolved multiplexing technologies such as mIF into clinical practice based on their intended uses. Modern approaches advocate for use of mIF for exploratory clinical sample analysis as a more straightforward methodology. As such, procedural integrity is key to enabling a more comprehensive and locked-down assay, analysis, and reporting strategy—under appropriate quality control (QC) processes—necessary for regulatory device submissions.

## 2 mIF companion diagnostics product concept

A goal of companion diagnostic (CDx) development programs is simultaneous development and regulatory approval of drug and test in parallel coordinated tracks, ensuring that when the drug is approved with test indicated and required as per the drug label, the test is also readily available. These parallel development processes converge in the form of a clinical trial to validate the CDx test’s effectiveness to identify patients by the presence or absence of a biomarker and, in turn, determine therapeutic efficacy within a population of patients selected (or not) to receive a particular drug.

The Food and Drug Administration (FDA^4^), EMA (European Medicines Agency^5^) and ICH (International Council on Harmonization^6^) have issued similar draft and final guidance or reflection documents that assist in the development activities and approval routes for *in vitro* companion diagnostic (IVD CDx) tests, including medical device clinical investigation design and Investigational Device Exemptions (IDE^7^) to allow the test to be used in early- and late-phase studies to collect safety and effectiveness data. These documents advise experimental design, ranging from exploratory feasibility studies of a biomarker for its proposed intended use to prospective or retrospective clinical trials to confirm clinical utility.

These statements above apply specifically to CDx; there are no corresponding written rules for complementary diagnostics, the definition of which is currently by usage (Milne et al., 2015; Scheerens et al., 2017; Jorgensen, 2020).

### 2.1 Design principles

A successful IVD CDx strategy requires products to be developed, verified, and validated using design control principles in an organization with a quality system in place and certified to develop and manufacture medical devices. As implied, this is the following of a well-documented controlled process to develop a device, as typically covered within a product concept document describing user needs and market requirements.

The initial stage of the process is the development of a prototype assay, when components of the assay become formalized, with decisions being made by the CDx developer about the technology platform.

The product concept document defines the assay type and a set of reagents, sample preparation, instrumentation, software, and test scoring/interpretation guidelines that will be validated together as a locked device or platform. The development of this assay or device/platform occurs in stages that are aligned with the drug development pathway and clinical trials (“drug-diagnostic co-development model”).

Platform choice is a key decision point in setting the specifications of an assay in this prototype stage. Intellectual property needs are defined, with a clear understanding of this and other risks. As an example, the workflow might need to be compatible with common and custom laboratory information management systems. For image-based analysis, data processing workflows may also need to support remote viewing and annotation and be capable of handling the scale and size of images and data sets in a HIPAA-compliant manner (Health Insurance Portability and Accountability Act of 1996^8^). Image analysis software is typically custom and locked down with few adjustments, since test operation needs to be as reliable, automated, and as simple as possible to avoid errors and for consistency of results. Although research-oriented software is useful for discovering effective analysis algorithms, these are usually set aside after an analysis algorithm has been selected for CDx.

Once the system solution is determined, the development process moves to design verification—the stage where design output is tested against design input. Design input is a set of detailed analytical conditions that must be achieved if the final device is to meet its intended use and be fit for purpose. Verification demonstrates that the assay, often referred to as an Investigative Use Only (IUO^9^) test, will meet all performance criteria and fulfill the product requirements, after which the design will be locked.

To that point, while the most common IVD CDx assay output is likely to be binary (i.e., positive or negative), at least with respect to a predefined cutoff profile, it is not a requirement. The collective use of multiple biomarkers offers some level of flexibility during IVD CDx development that shapes performance characteristics for a specific clinical application. Multivariate tests such as mIHC can provide several quantitative outputs that support more nuanced decision making, perhaps values of multiple variables combined using an interpretation function to yield a single, patient-specific result.

In IO, the process of developing a multiplex panel usually starts with biomarker selection by a scientific team comprised of immunologists, cancer biologists, and technical specialists, focused by one or more hypotheses related to the drug and/or target biology. These exploratory-use assays typically support promising hypotheses for predicting response. Once exploratory work is complete and correlations are determined empirically, the multiplex panel may be reduced to only those markers necessary to achieve an outcome: marker reduction is a statistically driven process that strikes the right balance of simplicity and predictive power.

Feedback should be sought from the FDA for clinical studies that began with an assay version different from the one ultimately filed with the agency, perhaps as simple as a manufacturer change, to another using a different IVD CDx device configuration (assay, instrument, or analysis) entirely.

In the former, an analytic bridging study is designed that shows earlier and current versions of the test perform identically. In the latter, as test design or manufacture has been changed significantly, the bridging study may include re-testing all samples run with the prior version of the clinical trial test with the final validated test. Such bridging strategies should be avoided as much as possible, however, where unavoidable, built thoughtfully into trial sample, consent, and test planning for accommodating sample re-testing requirements.

### 2.2 Validation

A product concept document defines the prototype assay and analytic plan that can be used to demonstrate proof-of-concept for the technical and clinical validity of the final IVD CDx. Loosely speaking, it identifies the link between biomarker status and the clinical outcome following treatment with the investigational drug and thus confirms a proposed biomarker hypothesis. Feasibility studies focus on establishing a minimum accepted level of analytic performance that include, but are not solely limited to, precision and reproducibility, accuracy, sensitivity, and specificity.

Normally, development and production activities for a prototype IVD CDx assay are first completed to a standard as required by CLIA (Clinical Laboratory Improvement Amendments^10^) or accredited professional organizations such as CAP (College of American Pathologists^11^). CLIA does not specifically use the term “validation” but refers to “establishment of performance specifications.”

Guidelines to assist in establishing performance specifications have been published by the Clinical and Laboratory Standards Institute (CLSI^12^) and the International Organization for Standardization (ISO^13^)—e.g., ISO 15189 (Standardization Medical Devices—Quality Management Systems - Medical Laboratories—Particular Requirements for Quality and Competence^14^). Regardless, actual practices remain variable. Some states (e.g., New York^15^) also have state health laboratory organizations that impose specific requirements that are comparable with or more stringent than CLIA regulations.

Even if a manufacturer does not intend to distribute the final device but makes it available from a single location, as a Laboratory Developed Test (LDT), certain performance specifications still must be met that would ensure assay consistency among users, as per CLIA and CAP guidelines. A manufacturer may also seek regulatory approval to obtain IVD (*in vitro* diagnostic device) clearance from the FDA, in a process called single-site premarket approval (ssPMA^16^) to obtain a Class III medical device approval. This approval is subject to many of the same quality system requirements as any IVD manufacturer and the level of analytical performance to an IVD CDx device employed as an aid in identifying patients for treatment.

A critical aspect of any assay development and validation process is selecting a training set that is representative of the real-world samples being collected in the clinic and ensures the test has appropriate analytical sensitivity and specificity as compared to the expected distribution and biomarker prevalence. Alternative methods for measuring analytes (orthogonal testing) further establish specificity, and health authorities often require such data.

Analytic validation studies are used to demonstrate real-world test precision, or repeatability (single site/single run/single test operators) and reproducibility (multiple sites/multiple runs/multiple test operators), chiefly at the limit of detection (LOD) or at a clinical decision point. This approach confirms the capability of the test to distinguish between positive and negative samples with consistency. The cutoff defines the positive test result and infers the prototype assay may be a clinically useful treatment decision tool, which will divide the intended patient population into likely responders or non-responders to the investigational drug.

## 2.2.1 Clinical decision points—Cutoff values

The classic CDx paradigm is that cutoff values are determined from early phase (I-II, mostly II) clinical studies based on retrospective data analysis after which a clinical threshold can be prospectively validated in a subsequent late stage (III) trial.

Breaking with this, cutoffs are more frequently being chosen and used prospectively for patient selection in earlier phase clinical trials before they are completely validated. This approach introduces risk as trial outcome might be determined using a cutoff that may not be fully informed by earlier studies (drug development risk) or using a CDx assay that may not have been fully validated for performance around the cutoff at the time of enrollment (diagnostic risk).

To the latter point, when initial clinical outcome data are available and test cutoff is defined, the validation set used to establish analytic performance for the new device includes samples below, above, and at the cutoff (each) to ensure the prototype IVD CDx test returns an accurate result. The absence of specimens with clinical outcomes would require that the cutoff selection be informed by biomarker prevalence and/or intensity measurements.

**FIGURE 3:**
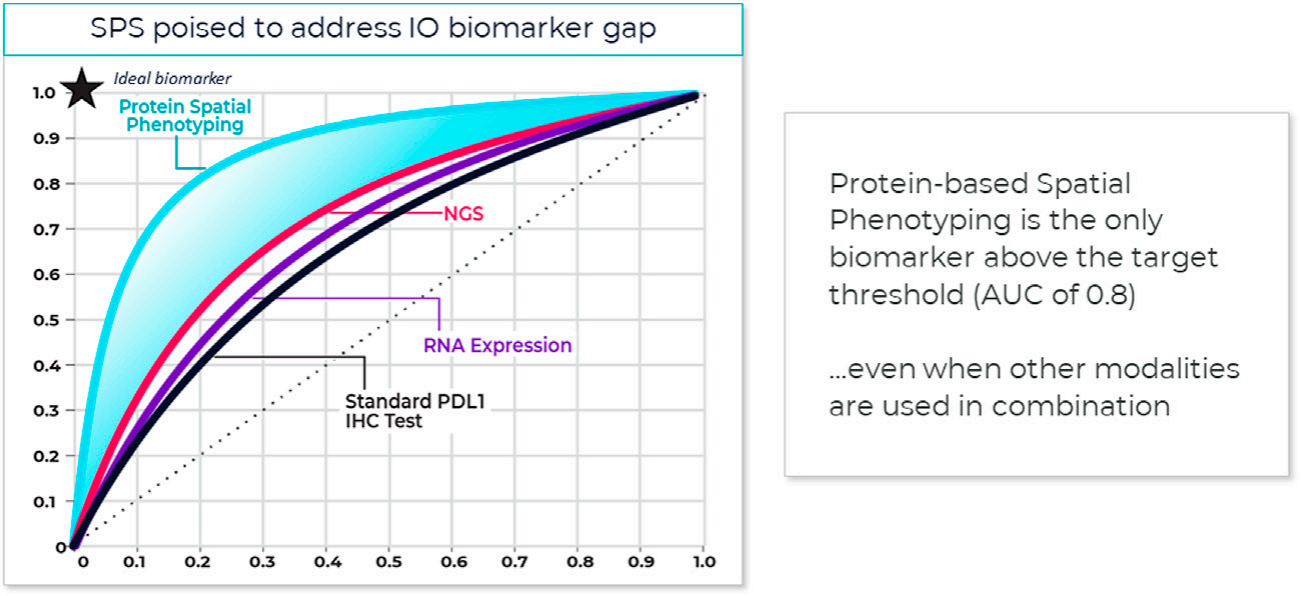
**Spatial phenotyping provides the highest predictive value**. The standard for comparing the diagnostic accuracy of biomarkers is the receiver operating characteristics (ROC) curve (Nahm, 2022). This is a plot of sensitivity versus 1-specificity across a range of cut points for a biomarker. The plot illustrates how well a model can discriminate or separate the cases and controls. The area under the curve (AUC) has a value between 0.50 and 1.0, with 0.5 indicating no discrimination and with 1.0 indicating perfect discrimination (Simundic, 2009). An excellent biomarker has an AUC of 0.8 or higher. The AUC of the ROC curve reflects the overall accuracy and separation performance of the biomarker (or biomarkers) and can be readily used to compare different biomarker combinations or models (Sanghera et al., 2013).

One method that has proved useful in selecting cutoff values is the receiver operating characteristic (ROC) curve (Figure 3) (Nahm, 2022). Sensitivity (true positivity) is plotted against 1-specificity (false positivity) for different preliminary selected cutoffs. Subsequently, the area under the curve (AUC) can be calculated for the different cutoff points, which serves as a general figure of merit for predictive power. A cutoff can be selected based on goals for the assay, such as achieving a satisfactory balance of low false negative rate while providing clinical utility or using standard approaches. For example, maximizing Youden’s J statistic identifies the point on the ROC curve closest to the upper left corner of the plot (Simundic, 2009).

Reducing a set of markers to a fit-for-purpose clinical assay is a biostatistical process that might include rank ordering measured biological parameters according to individual AUC, selecting top ranked parameters, to avoid data model overfitting, and defining a scoring equation that includes selecting thresholds to identify patients with a higher likelihood of responding to therapy. This can mean that some markers in an exploratory multiplex panel not associated with top-ranked parameters are dropped from the test or from the analysis.

**FIGURE 4:**
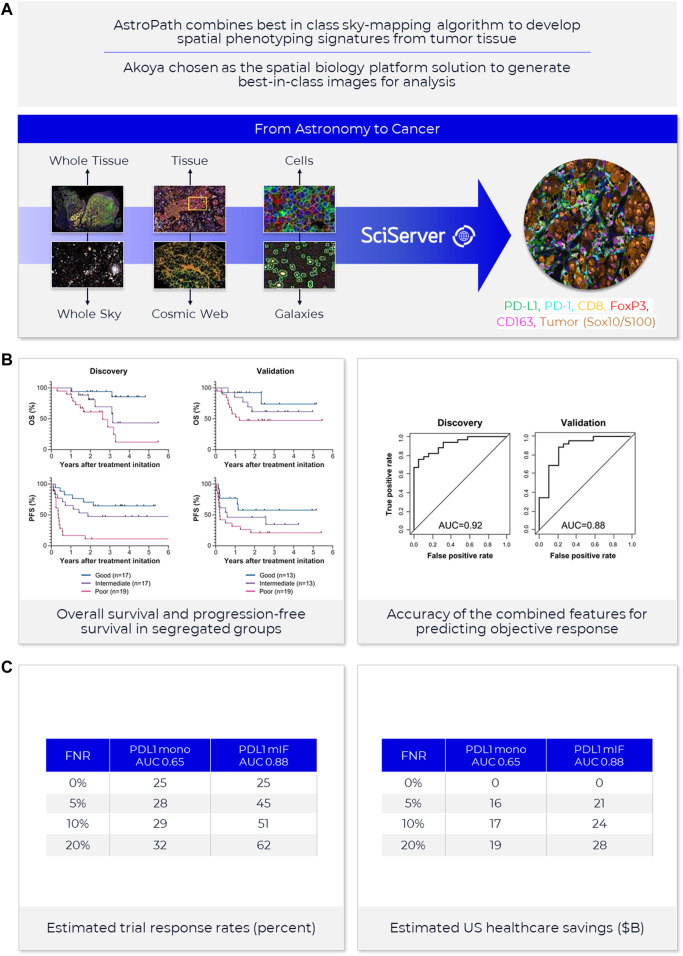
**AstroPath—astronomy meets pathology, a novel approach to developing SPS that is highly predictive of PD-1 therapy**. **(A)** AstroPath is a sky-mapping algorithm developed at Johns Hopkins University to stitch together millions of images of billions of celestial objects, each expressing distinct signatures. Applying those principles to mIF imaging using Akoya’s spatial biology platform, John Hopkins researchers were able to develop SPS. **(B)** Using a six-plex (PD-1, PD-L1, CD8, FoxP3, CD163, and Sox10/S100) Akoya mIF panel, the team at John Hopkins University were able to develop 41 combinations of expression patterns and map relatively rare cells. This multifactorial analysis was used to study 10 features for predicting objective response in melanoma patients after immune checkpoint—blocking therapies, ranked in decreasing order of predictive value. Patients could then be assigned to one sof three groups: poor, intermediate, and good prognosis, with characteristic cell co-expression phenotypes detected by the mIF assay. The TME from patients with poor prognosis was characterized by high densities of tumor cells and CD163+ cells that lack PD-L1 expression, irrespective of whether other immune cells were present. The area under the curve (AUC) values were assessed for the 10 features for both the discovery cohort and the validation cohort and showed an excellent accuracy in predicting objective response (AUC of 0.92 and 0.88, respectively) (Berry et al., 2021). **(C)** AUC implications for trials and healthcare costs, with Build Model based on publicly available data on response rates for leading IO tumor indications. Shown are estimated trial response rates and United States healthcare savings if using PD-L1 monoplex (AUC 0.65—Lu et al., 2019) or PD-L1 mIF (AUC 0.88—Lu et al., 2019) for patient selection with false-negative rates of 0%–20%. Publication copyright for Berry et al., 2021 is governed by a CC BY 4.0 License.

Sufficient AUC needs to be the overarching goal, since it drives trial and drug success. Based on an internal model assessing trial success rates and health economics considerations, a reasonable AUC target for predictive tests in IO should be 0.80 or greater (see also Figure 4).

Recent meta-analysis of anti-PD-1/PD-L1 therapy data pooled from 50+ studies spanning 10+ tumor types and 8,000+ patients used ROC and AUC to examine the predictive value of single-marker IHC (PD-L1), tumor mutational burden (TMB), gene expression profiling (GEP) and PD-L1 mIHC/mIF. The results revealed that the category including mIHC/mIF performed significantly better compared to the other three assay types (Lu et al., 2019). For instances, such as mIF, the number of positive outcomes may be increased with better patient selection and stratification, and the design, size and cost of the study reduced due to fewer patients in the clinical trial (Olsen and Jorgensen, 2014). Thresholds for clinical trial patient selection assays in phase I or II used are typically set according to such exploratory data but can be subsequently adjusted once the clinical trial assay data is analyzed to establish locked thresholds for pivotal trials.

### 2.2.2 Clinical decision points–Study design

Though the discovery of important predictive biomarkers is largely a medical/clinical or biological problem, statistics plays a vital role in all areas of precision medicine, ranging from study design to analysis. It is fair to state that the analytic validation of multi-variate tests will require more refined study designs than for single-plex IHC assays (Campbell, 2021).

Among other recommendations^17^ ^,^ ^18^ ^,^ ^19^ ^,^ ^20^, CLSI describes a study design^21^ for IHC assays using specific numbers of validation samples in replicate (or minimum data points) that can be used to estimate test precision. Any type of study design typically allows the calculation of within-run, between-run, and between-day differences, readout as coefficient of variation (CV) for the assay, as an example. Sample availability at or near a cut-off poses a challenge of sufficient statistical power if only small numbers of validation samples can be identified.

### 2.2.3 Clinical decision points–Economics

To provide a sense of the impact of achieving more precise, selective and clinically- or economically-actionable test (Sanghera et al., 2013), including those using ROC and AUC to examine predictive values for different thresholds of monoplex versus multiplex tests, our own estimates based on public data^22^ ^,^ ^23^ on incidence rates and therapy response rates in immunotherapy-eligible indications suggest that increasing AUC from 0.65, typical of PD-L1 IHC tests, to AUC 0.88 as shown for PD-L1 mIF (Lu et al., 2019) would double response rates in clinical trials and potentially save the United States health system in the $10–20B annually (Figure 4).

Practices that tease out which test methods are economically actionable are poorly characterized. More exact economic assessment of mIF as a cost-effective treatment is advised and appropriate for informing decisions for where only patients with economically actionable results will receive targeted treatment (Haslam et al., 2020).

### 2.2.4 Clinical decision points—Intended use

Frequently at this stage in IVD CDx device development, the various benefits of the assay/drug pairing have yet to be demonstrated, testing costs and reimbursement rates are not considered, and assay development is primarily focused on analytic performance, such as precision and reproducibility. Meanwhile, assay results are used for exploratory purposes and typically not for trial patient selection. If the assay is intended to be used for patient selection, and might pose significant risk to patient safety in the United States, then filing of an IDE is necessary and the activities supporting analytic performance must be approved to ensure no clinical investigation delay.

Additionally, studies investigating the use of these candidate diagnostics for a candidate therapy must be designed to follow FDA regulations pertaining to investigational new drugs (IND^24^). To streamline the process of determining when an IDE is needed, comprehend approval requirements, and understand the development process in general, device manufacturers often use a pre-submission process (Q-Sub^25^) to initiate a dialogue between the FDA and the manufacturer on the device’s design and intended use.

Q-Subs can help coordinate interactions among FDAs different centers [for Drug Evaluation and Research (CDER^26^), for Biologics Evaluation and Research (CBER^27^), for Devices and Radiological Health (CDRH^28^)] and is important particularly for drugs that are seeking accelerated approval (e.g., Breakthrough Device Pathway^29^) so that device approval delays do not impact the drug timeline. This is particularly true with novel devices and technologies such as mIF whose clinical utility has yet to be established but might provide clinically meaningful advantages over existing/predicate devices.

For mBF and mIF, as automation will almost heavily underpin the analytical robustness of the final device, the performance of the assay on a specific instrument must be described in detail, including operational specifications and calibration. As CDx assays are viewed by regulatory authorities as integrated, fixed systems with strictly defined component. For example, a specific autostainer model and version(s) of operational/analytic software also become part of the device and are collectively locked-down by version control to prevent changes to the device.

Device labeling, normally discussed as part of the Q-Sub process, determines how the CDx should ultimately be used to support the indicated uses with single or multiple drug or biological products. The intended use statement drives all subsequent performance attributes covered under the verification process. Any change from the established system components or parameters as described in the product package insert would be considered a deviation from the approved use of the assay and would thereby invalidate the test results.

### 2.3 Verification

In transitioning from prototype assay to IVD product, subsequent work is performed in a controlled manner under Good Manufacturing Processes (GMP^30^) also covered by 21 CFR 820 (United Ststes Quality System Regulations^31^) or ISO 13485:2016 (Standardization Medical Devices—Quality Management Systems—Requirements for Regulatory Purposes^32^). Certification to the ISO standard has become an essential requirement in the European Union (EU) and Canada.

21 CFR 820.30 covers design transfer to ensure device design and design outputs are verified as (transferred into) suitable production specifications. Product attributes include QC and performance specifications for raw materials, manufacturing process validation, in-process and final release QC, requirements for shipping conditions and stability determinations of raw materials, intermediate batches, and the final product(s).

ISO 13485:2016 certification, specifically and particularly, ensures the design, development, production, distribution, and installation of medical devices, such as software or instruments, meets industry requirements in the international market since different countries might have different quality standards.

In either instance, all verification activities are properly documented in a design history file (DHF) using a set of design input requirements that match design output specifications. Inherent to the process is the requirement for periodic design review stages when the device’s performance is assessed against the requirements, although the subsequent analytical verification of the assay provides objective evidence that the design specifications have been met according to the intended use.

Key performance attributes being assessed during verification include analytical sensitivity, analytical specificity, precision, robustness, and stability.

### 2.3.1 Clinical decision points—Robustness

Not covered previously, robustness studies (flex- or guard-band studies) assess the ability of the IVD CDx to remain unaffected by small and deliberate changes to assay parameters. These provide an indication of its reliability to function correctly under varying conditions of proper and improper use, ensuring that slight variations encountered in the operation of the test during normal use do not affect results.

Notably, key attributes of a well-designed product concept document will also include understanding and verification of the inherent (biological or otherwise) variability in sample collection and handling methods, processing, and stability. For IHC tests particularly, there is a strong appreciation for the impact of preanalytical variables (e.g., warm/cold ischemia time, fixation time and post-fixation tissue processing, FFPE microtomy, and slide storage conditions, etc) on technical aspects of testing to ensure analytical trueness.

Particularly for FFPE biopsy samples, the quality of the sample is a major determinant for test success, so ensuring the assay is robust enough for low-input test material is important, provided there is established QC criteria for identifying potentially degraded samples (Lazcano et al., 2021). An added convenience of (some) high-level multiplexing platforms may be the ability to include the evaluation of standard markers to verify tissue quality.

Beyond only a few biomarkers (e.g., ER, PR, HER2—Grillo et al., 2016; Ehinger et al., 2018), rigor and guidelines covering sample fixation, processing, and storage are not well defined—which is less problematic when local control of the clinical trial sample collection process is in place. However, the ramifications are magnified for a global clinical trial that might limit the utility of the test as a reliable diagnostic platform.

Routine histopathology plays a critical role, with optimal tumor tissue fixation reagents, fixation time, and age of blocks and sections, as well as method of storage being critical for high-quality assay performance. An estimated 10%–20% of biopsies collected are unfit for diagnosis due to errors in pre-analytic steps, requiring new samples to be collected (Ferry-Galow et al., 2018; Bhamidipati et al., 2021).

Investigation of the stability of the assay components is conducted with a final, commercial-ready version of the assay under quality system guidelines and performed on at least three pilot production lots. Preliminary shelf life may be established in an accelerated stability study, but final shelf-life requires a study conducted in real time that incorporates scenarios reflecting the intended transport and standard on-site use of the assay. The stability of the intended analyte in samples must also be determined, also through a real-time stability study with slide-mounted FFPE sections. While such results guide appropriate labeling for the validated IVD CDx, they also identify potential risks for routine laboratory use.

In addition, verification ensures risks associated with the final IVD CDx device are being categorized for severity and probability, and mitigation strategies identified. For CDx, risks are normally associated with the likelihood and severity of an incorrect result, and impact to patient safety. Ideally, the prototype assay used to demonstrate feasibility of the CDx concept will de-risk later-stage work by identifying and then providing mitigating strategies for biological, technical, logistical, and material challenges.

### 2.3.2 Clinical decision points—Quantitative image analysis methods

With slide-based IHC tests typically assessed visually, a major risk is intra- and inter-observer variability (Marchevsky et al., 2020). Quantitative image analysis methods should improve the consistency of scoring and interpretation and may also more accurately identify patients with low biomarker expression that challenge human perception. It should also enable cross-site comparisons subject to international proficiency testing or external quality assurance (IPT/EQA) examination, and eventual clinical translation as biomarker discovery platforms or standard diagnostic tests.

Panels of IHC markers are used typically as an adjunctive to standard pathological diagnosis (Painter et al., 2010), and help answer questions relevant to a specific clinical scenario. While a pathologist is capable of interpreting histological staining patterns using biological context, and naturally compensating for staining and tissue variability or artifacts to assess relative protein expression levels, they are not as well suited to making quantitative measurements of expression level or cellular percent positivity (e.g., Troncone and Gridelli, 2017; Tsutsumi, 2021; Butter et al., 2022). Conversely, IA is dependent on digitizing and measuring specific image features that relate to biology in question and are therefore very reproducible. Areas of vulnerability exist in the subtle changes of expression level and staining patterns, as well as specific image features/classifiers: color deconvolution, section thickness, thresholds, and parameters used for cell and feature segmentation (Kohlberger et al., 2019).

Since pathologist assessments are often the gold standard for regulatory approval of IHC assays, verification plans should be designed accordingly to accommodate these inherent differences. Moreover, the practicing pathologist engaged in the validation/verification processes is usually an expert who is or becomes particularly proficient in scoring the assay under study, and not one who would be scoring in clinical practice. Beyond only a few biomarkers (e.g., 510(k) FDA-cleared algorithms for HER2^33^) has an IA application received regulatory clearance for use as an aid to pathologists. Until IA is routinely validated and accepted as a component of IHC-based CDx tests, reader variability will remain a critical consideration in the verification of slide-based assays.

A newly described and innovative multidisciplinary approach, called AstroPath at the Johns Hopkins University (Berry et al., 2021) (Figure 4) leverages the principles of immunology, pathology, computer science, and astronomy to lay the foundation for rapid, efficient biomarker discovery through IA. This novel approach turns discovered predictive signatures into analytically and clinically validated assays.

The approach delivers on two important translational goals—creating an imaging and data analysis pipeline that is highly reproducible and accurate for classifying critical tell-tale cells in the TME, while simultaneously storing and making accessible for data mining vast data sets that come out of studies. The database is highly optimized for rapid data querying and integrated with a cloud-based digital pathology interface.

AstroPath, based on the PhenoImager^®^ workflow of Akoya Biosciences^34^, uses celestial object mapping algorithms for massively scalable, quantitative spatially resolved single cell analysis of the results from mIF assays in a TME as part of an end-to-end pathology-centric workflow.

## 3 Multiplexing—PhenoImager

The PhenoImager solution from Akoya Biosciences is a complete end-to-end workflow that supports research and clinical trial objectives for mIF (Table 2). The technology includes reagents and protocols for automated and manual mIF staining, multispectral imaging instruments capable of field-of-view (FOV) and whole slide image (WSI) acquisition, as well as software applications for basic and complex image review and analysis including data reduction using R-script packages.

**TABLE 2:** Intended use performance requirements for spatial biology using mIF and MSI. Translational attributes of mIF (PhenoImager assay and workflow) for biopharma discovery/exploratory trials, clinical research/trial inclusion and standard-of-care/diagnostics.

Exploratory		Trial inclusion	Diagnostic
Early Phase (I/II)	Example data use	Trial Inclusion (I/II/III)	Therapy Decisions
Hypothesis Generation, incl. MOA		Cohort Expansion	Transfer to IVD Partner
Profiling—Biomarker Prevalence		Regulatory Submission	Regulatory Submission
RUO-level validation (may be CAP/CLIA or GCLP^1^ levels)	Requirements	CAP-level validation	LDT: CAP and CLIA levels
		CLIA registration	IVD: FDA clearance (PMA, ssPMA)
		GCLP^a^, ISO 15189	GCLP^a^, GMP, ISO 13485
Antibody, Specificity/Accuracy^b^			
Yes	Cell lines (endog, transfected)^c^	Yes	
Optional	sh/siRNA or CRISPR^c^	Preferred	Yes
Optional	Peptide/protein competition^c^	Optional	Yes
Yes	RNA ISH	Yes	
Optional	Orthogonal (e.g., WB, RT-PCR)	Preferred	Yes
Optional	Normal TMA^d^	Yes	
Can proceed if “NO”	Antibody, freedom-to-operate	Can proceed if “NO”	Must be “YES”
mIF Analytic Development			
Same	Mono-mIF concordance^e^	Qualitatively/quantitatively comparing staining of each individual target as a monoplex (DAB and IF) to each same target in mIF panel using serially adjacent tissue sections	
Same	Drop Test and Interference^f^	mIF panel drop controls to determine antibody and/or fluorophore interference if markers are co-expressed using at least 2 different tissue samples and 8* serial sections of each (*for 6 marker panel)	
Same	Signal Intensity^g^	Normalized counts of 10–30 for all Opal fluorophores, except Opal Polaris 780, where the recommended range is 1–10 counts	
Same	Dynamic Range^h^	Signal-to-background (SNR) ratio of 10+ for adjacent channels	
Same	Opal Signal Balance^i^	Ratios of signals between neighboring channels of 3:1 or less	
Same	Opal Cross-Talk^j^	Residual cross-talk of ≤1% to ensure minimal interference with IA	
Analytic Performance (robustness)			
Yes (3–5 slides in 1 run)	Precision (intra-run)^k^	Yes (3–5 slides in 1 run), tissue samples preferably Neg L M H or −/+	
Yes (3–5 slides in 2 additional runs)	Reproducibility (inter-run)^k^	Yes (3–5 slides in 2 additional runs), tissue samples preferably Neg L M H or −/+	
10–20	Sensitivity/Prevalence (sample#)	40–50^m^	
Contextual (assay type), at least 2 operators and 1 instrument)	Inter-instrument/user	Contextual (assay type), at least 2 operators and 1 instrument)	Contextual (study type), at least 3 operators and 3 instruments)
Optional	Epitope stability	Preferred (from at least 3 timepoints, and up to 6 months	Yes (from at least 3 timepoints, and up to 12-18 months)
No	Reagent stability	Optional (Yes, if diluted RTU and stored)	Yes (real-time, accelerated and extended stability)
Same	Photostability^l^	Scanned repeatedly over the course of 6 months with <10% loss of signal	
Preferred	Antibody Lot/Lot QC	Yes (ISO standard quality)	Yes (at least 3 cGMP Lots)
Can proceed if “NO”	Antibody, freedom-to-operate	Can proceed if “NO”	Must be “YES”
Algorithm Performance and Report			
Fit-for-purpose algorithm (for use only with sample set/specific indication used during the development of algorithm)	Example data use	Fit-for-purpose algorithm (for use only with sample set/specific indication used during the development of algorithm)	General purpose algorithm (broad enough to use with an independent validation set) or fit-for-purpose algorithm for a specific indication
Preferred	Pathology Input	Yes. Manual annotations of tumor region, exclusions, etc	
Initial training on 5–10 samples, 3–5 FOV per sample. Performance confirmed by senior scientist and/or pathologist. Algorithm re-trained when needed	Training^n^	Initial training on 5–10 samples, 3–5 FOV per sample. Performance confirmed by a pathologist. Statistically powered validation test set. Comparison with pathologist annotation, review, and sign-off. After successful validation, if issues arise requiring retraining, re-validation is required	
Quality control by visual review of WSI (≥10% fields processed) or FOV (≤20 per slide) being processed, including run batch stain controls	Image QC Process^o^	Quality control by rigorous quantitative and pathologist visual review of a subset of areas from WSI (for ≤6 markers) or FOV (typically for ≥7 markers) being processed, including run batch stain controls	
Optional	Locked Algorithm	Preferred	Yes
Same	Readout	Cell phenotype (frequency, density, proximity, compartment), upset plots, tissue maps	
No	Report incl. Sign-Out	Yes. Pathologist reviews and approves/flags image analysis results	

Assumptions and Notes

^a^ Notes: Good Clinical Laboratory Practice (GCLP) is a set of standards that provide guidance on implementing principles of Good Laboratory Practice (GLP) and directives within the ICH, Guideline for Good Clinical Practice (GCP) to the analysis of samples from a clinical trial. GLP is a quality system that covers the organizational process and the conditions under which studies are planned, performed, monitored, recorded, archived, and reported. GCP is an international ethical and scientific quality standard for designing, conducting, performing, monitoring, auditing, recording, analyzing, and reporting clinical trials that involve the participation of human subjects. By combining the GLP and GCP sets of guidelines, GCLP ensures the quality and reliability of the clinical trial data generated by laboratories.

^b^ Assumptions: Reviewer is a pathologist who is familiar with the target and can confirm the associated biology and staining pattern.

^c^ Assumptions: Cell lines can be identified and acquired showing endogenous expression or no expression of the target, or cell line can be transfected—preferably at different levels—with the target of interest, and/or with different but related members of the same protein family to further demonstrate specificity. Assumes also that target can be expressed as purified protein and sh/siRNA or CRISPR targeting is possible (cell line and/or gene).

^d^ Notes: Normal TMA, refers to a human tissue array used to test normal tissue specificity of antibodies that is designed in conformance with FDA guidelines and requirements for tissue cross-reactivity studies for IVD certification that contains 30+ types of normal organ, each single core case from at least three individuals.

^e^ Assumptions: Targets in the mIF, are paired with the appropriate Opal fluorophores e.g., the brightest Opal fluorophores with the weaker expressing proteins, and *vice versa*. Various correlation coefficients (e.g., Pearson, Kendall, Spearman, Lin, Interclass), ideally reporting above 0.9 or 90% positive concordance, are acceptable.

^f^ Notes: Each marker detection is not affected by the presence of other markers. Interference is established using a drop test to identify markers involved in an “umbrella effect,” a term commonly used to describe when a previously applied marker impedes the application of an additional marker that colocalizes with the first. This is commonly seen in mBF, assays if using DAB. With TSA, inhibition of antigen recognition can be of a mechanism other than steric hindrance (umbrella effect) and result from the depletion of sites for activated tyramide binding. Both are important to acknowledge in instances when using TSA, if ≥ 2 markers of interest are in the same cellular compartment.

^g^ Notes: These ranges support reliable and accurate data analysis. Reliable data can still be obtained when signals are as low as a few counts or as high as 50 or more counts, but risks are higher for cross talk issues.

^h^ Notes: SNR calculated by dividing the average signal intensity of the top brightest 20 cells by the average signal intensity of the weakest 10% of cells. Typical ratios are 100+ with high-performing antibodies; SNR, above three can still provide analytical value.

^i^ Notes: Most relevant for using Opal fluorophores in the 520, 540, and 570 channels in the same panel (classic: Opals 520, 540, 570, 620, 650, and 690). With MOTiF, which replaces Opal 540 with Opal 480 and Opal 650 with Opal 780, the 6 fluorophores are more spectrally distinct.

^j^ Notes: There are two main sources of crosstalk; 1) instrumental crosstalk occurring when fluorescence signals leak from one channel to another due to imperfect filter optics or from inadequate crosstalk compensation algorithms; 2) staining crosstalk from actual fluorophore inaccurately labeling proteins on the sample resulting in residual fluorophores inadvertently binding to epitopes intended to be labeled by another fluorophore. It is very important to distinguish the two causes because resolving each is a different process.

^k^ Assumptions: Co-efficient of variation (CV) of 20% or less, preferably an upper %CV limit of 15%.

^l^ Assumptions: Slide stored at room temperature in dark. Camera exposure times should ideally be in the msec range for each fluorophore. This signal level translates to slide scan times of approximately 15 min for a 7-color assay at Magn. ×20 (∼0.5 µm × ∼0.5 µm pixel size) for a typical resection biopsy with area of 1.5 cm × 1.5 cm.

^m^ Notes: Capacity to detect cells with full range of marker expression. For predictive marker assays, labs should test a minimum of 20 positive cases and 20 negative cases. If the Lab Medical Director decides that a validation set of ≤40 cases is sufficient, they will need to document the rationale.

^n^ Notes: Accuracy to count cells should be based on single markers. Cell classification accuracy using mIF, according to any marker co-expression, should be as provided by other single-cell platforms, while ensuring no signal contamination from neighboring cells. Cell segmentation can be challenging, complicated and may not always be robust across different cell types and/or due to staining variability or non-uniformity.

^o^ Note: Some WSI,analysis algorithms do not process whole slide images as a monolithic image, but rather in a field-based fashion with data from multiple FOV reduced to a single output afterward.

The workflow utilizes tyramide signal amplification (TSA) to intensify and stabilize fluorescent signals (e.g., Stack et al., 2014; Surace et al., 2019; Francisco-Cruz et al., 2020; Parra et al., 2020; Boisson et al., 2021). Different TSA-linked fluorophores are available under the Opal trademark. Peroxidase-catalyzed oxidation of TSA dye substrates by HRP creates free radical tyramide species that locally and covalently bind to tyrosine, tryptophan, histidine, and cysteine residues of proteins in tissue sections (Bobrow and Moen, 2001). This HRP-mediated amplification strategy, like the industry-wide standard HRP/DAB method (Nakane and Pierce, 1966), enables the detection of low-level target expression by elevating FL signal above background tissue AF.

A typical TSA-based workflow requires sequential detection of primary antibodies, with heat, pH or other methods (e.g., salt, chemical) used to strip primary and secondary complexes from the tissue section, leaving the covalently-bound fluorescent tyramides at the site (labeling radius ∼200 nm) of primary antibody binding. While TSA-based workflows are iterative and therefore multi-step, with potentially 12–18 h run times, repeated exposure of tissue to high temperature or pH conditions (or other) are a consideration for occasional more-labile antigens. But this issue is readily addressed through determining marker staining order. In fact, repeated heating steps are useful to achieve more complete retrieval of many important functional markers, such as PD-L1 and Ki67.

Tyramide conjugation is used by multiple platforms, including for BF mIHC using TSA-chromogens that are HRP substrates with narrow spectral signatures (Morrison et al., 2020). Despite this, implementation of mBF in clinical trial settings using greater than three biomarkers is challenging because of overlapping spectral signatures of each BF dye. Another limitation is that dynamic detection ranges are hindered by the optical density of light-absorbing chromogens. This issue is less prevalent when using FL-based methods, which are additive and advantage of linear signal processing, whereas light is absorbed and scattered using BF chromogens (van der Loos, 2008), which is subtractive and highly non-linear, leading to difficulty in separating markers and accurate quantitation.

The most effective utilization of mBF has been when targets are each expressed on different cell types or with different subcellular localizations in one cell type (Rimm, 2006; Ilie et al., 2022), thereby systematically avoiding pixel-level interferences from overlapping BF dyes. The need to include a colored nuclear counterstain, which may have broad and variable absorption spectra, can also complicate downstream IA analyses.

Other benefits of utilizing a TSA mIF workflow are a flexible target detection strategy and automated IHC workflow that can be easily implemented in routine clinical care settings, even in those with modest technical resources (Hernandez et al., 2021; Laberiano-Fernandez et al., 2021). Currently available Opal fluorophores support up to 8-plex FOV staining (9-colors including a DAPI nuclear counterstain) as well as 6-plex 7-color MOTiF whole slide image acquisition of entire tissue resections to further support translational workflows. Opal fluorescent dyes are very photostable relative to conventional IF methods and provide optimum spectral separation across the visible wavelength range based on detailed models of total system spectral response covering the entire optical train of the imaging system.

The PhenoImager workflow using a multispectral imaging (MSI) system resolves each IF marker into accurate single component images by capturing spectrally resolved information at each wavelength within each pixel of an image. The most current iteration of the MSI system is the PhenoImager HT (formerly known as Vectra Polaris), paired with two visualization software programs: Phenochart and inForm.

Phenochart is a whole slide contextual viewer enabling annotation of BF and FL slides scanned on the PhenoImager HT for MSI capture as FOV or WSI. This program has a live unmixing preview of WSI to support assay development and confirm staining performance. In addition, simulated DAB views of each FL channel can be provided, as can overlay of histological stains such as H&E. While mIF is visually stunning, most classically trained pathologists are unaccustomed to reviewing IF images and interpretating anatomical and morphological features in this format.

Further, H&E severely limits the further use of the same tissue section for IHC purposes. Creating a simulated pathology view with removable fluorescent dyes that accurately mimic traditional BF H&E to enable section re-use with no decrease in mIF performance^35^ is a benefit. This approach also allows the assessment of tissue quality before antigen retrieval while supporting the translation of mIF methods into clinical standards of care.

inForm is a software analysis package that integrates MSI and IA to spectrally unmix and isolate multiple FL signals and remove background AF (Mansfield et al., 2008). The software can detect different tissue architectures using an ML-based neural network pattern recognition function to segment individual cells based on DAPI and other markers in membranous and cytoplasmic regions. Moreover, it can phenotype cell types of interest based on signal intensity levels of one or more markers and/or cellular staining patterns using user-trained multinomial logistic regression algorithms.

inForm output data can be imported into a variety of other IA platforms for quantitative evaluation (e.g., HALO^36^, Visiopharm^37^, QuPath^38^). Open-source R-script packages, phenoptr^39^ and phenoptrReports^40^ that are publicly available on GitHub^41^ can further reduce high-dimensional single-cell data to staining pattern statistics, tissue region designations, and any other spatial parameter that might correlate best with clinical parameters such as response to immunotherapy.

## 4 Alignment of early biomarker development mIF platforms with CDx

High- and ultrahigh-plexing biomarker discovery platforms designed to cast a broad net of markers to support clinical hypotheses are often unsuitable for practical implementation and commercialization. If a technology is not commercially feasible, then a significant amount of biomarker signature data generated from early clinical trial activities cannot be translated into a fast-moved CDx competitive advantage.

A CDx development strategy that involves biomarker discovery on a higher plex platform should include a transitional bridging study that reduces the high-plex discovery panel to six or fewer of the most information-bearing markers and demonstrates equivalent or better analytical performance and ability to stratify patients into responders and non-responders.

Ideally, a CDx platform should be amenable to sample-in and score-out practices. The platform should have a significant global instrument footprint, protocols that require straightforward processing at the technician skill level, and the robustness of performance across geographic locations in terms of assay stability, reagent stability, supply chain assurance, and support from the instrument manufacturers for logistics.

CDx commercialization strategies should preferably be globally implementable to support robust implementation across various testing scenarios: local, centralized, or distributed testing in countries with reimbursable or universal healthcare.

Reimbursement is a key milestone in the path to clinical adoption and equally as important as demonstrating clinical validation and utility. Despite significant attention paid to personalized (i.e., precision) medicine, Health Management Organizations (HMO)/Insurers remain under significant pressure to address testing costs. For example, HMO reimbursement rates for multiplexed tests^42^ are low; there are three CPT (Current Procedural Terminology^43^) codes for reporting a qualitative IHC stain, and CPT directs not to use more than one unit of these for the same separately identifiable antibody, per specimen.

### 4.1 MITRE

**FIGURE 5:**
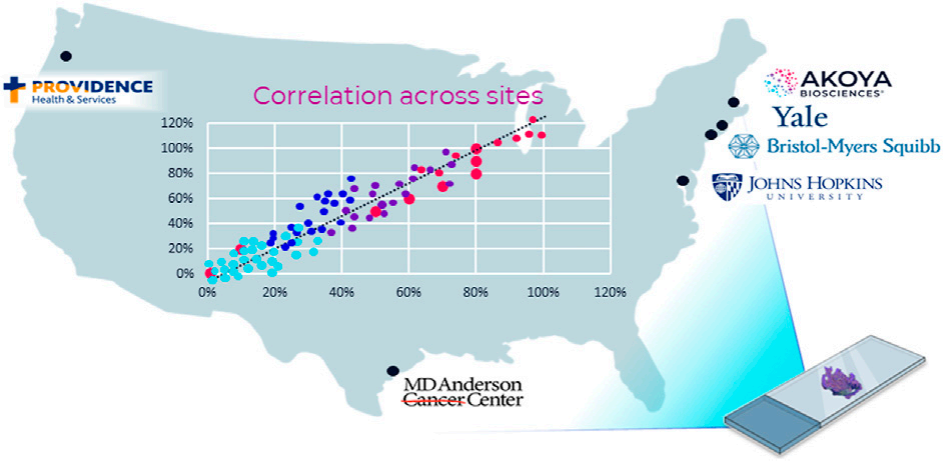
**MITRE: multi-institutional TSA amplified multiplexed immunofluorescence reproducibility evaluation**. Before mIF technology can potentially be translated into clinical practice, demonstration of the analytical validity and reproducibility of an end-to-end mIF workflow that supports multisite trials and clinical laboratory processes is vital. In this first multisite study, four leading academic medical centers, one pharma company, and Akoya Biosciences assessed the intersite and intrasite reproducibility of a 6-plex 7-color mIF assay. The results of the study show that the PhenoImager HT is the first spatial biology platform to meet reproducibility requirements for clinical applications (Taube et al., 2021).

Recently, a six-center inter-site comparison study with participants from biopharma and academia was conducted to demonstrate analytical performance across multiple institutions of a translational mIF workflow. The study was termed the multi-institutional TSA-amplified mIF reproducibility evaluation (MITRE) (Taube et al., 2021) (Figure 5).

Based on the PhenoImager HT platform, MITRE demonstrated the analytical reproducibility within and across all sites of an integrated workflow system for a mIF assay focused on the PD-1/PD-L1 axis also including CD8, CD68, FoxP3, and CK.

Further, the image analysis platform used in the MITRE study employed an AI/ML-based tool for segmenting and phenotyping cells, one which could be locked-down and shared to minimize inconsistencies. The locked-down capability also reduces discrepancies introduced by potentially varied practices related to local lab image analysis algorithm adjustments. Currently, effective interpretations of increasingly large datasets remain challenging, and analysis of such complex datasets has outstripped cognitive capacity.

### 4.2 Multi-dimensional data challenges

The push toward precision and personalized medicine has piqued interest in AI/ML approaches that structure, integrate, and interpret large datasets, to identify and validate highly predictive biomarkers to aid in clinical decision-making. This could ultimately lead to potential payoffs for patients and drug development programs. AI/ML approaches promise to parameterize and simplify vast tissue-based cellular data, consisting of protein co-expressions and spatial parameters, and to reveal easily interpretable models, such as SPS.

Tumor and immune/stromal cells comprising the TME represent a continually evolving ecosystem, characteristics of which are spatial and functional intra-tumoral heterogeneity. In cancer research, a recent explosion of ultrahigh-plexing biomarker labelling and imaging modalities probe the TME with 10s of biomarkers at cellular and subcellular resolution (Parra, 2021). Analysis approaches that also permit a more detailed analysis of the genome and transcriptome of single cells (e.g., scRNAseq) also fit within this emergent continuum but have achieved their successes at the expense of losing spatial context.

For high-dimensional single-cell phenotyping data, analysis methods that efficiently incorporate the inherent multi-parametric characteristics of such immunological data sets have been employed by the flow cytometry community for decades. Nevertheless, advantages of deploying such data processing tools within the histopathological space come with drawbacks, particularly the use of dimensionality reduction practices (e.g., tSNE, HSNE, UMAP - van Unen et al., 2017; Becht et al., 2018; Kobak and Berens, 2019) that disregard cellular subpopulations distinct in multi-dimensional space that might overlap in a reduced, two-dimensional space. A deep understanding of the programming (e.g., R^44^, MATLAB^45^, and Python^46^) behind these bioinformatic platforms is tremendously important in pathology to mine the depth and dimensionality of the data.

While deploying efficient methods to mine expansive data sets for optimum and actionable predictive signatures is a priority, assuring the accuracy of raw data going into data mining efforts is more important^47^. If a discovered signature relies on an unreliable measurement or parameter, the signature becomes unreproducible.

Another challenge of mining expansive multidimensional data sets for predictive signatures is over-fitting. Over-fitting occurs when the number of samples used as a basis for revealing a signature is not equal to or greater than the number of degrees of freedom in the multidimensional data set. Thus, combining focused hypothesis-driven tests on a subset of parameters and unsupervised clustering-based methods, such as AI/ML, benefits signature discovery and validation.

### 4.3 AstroPath

A relevant example of this is AstroPath (Berry et al., 2021) (Figure 4), which analyses, integrates, and models extensive (galactic) amounts of localized marker data that represent complex layers of biology and clinical medicine. Discovering and validating the optimum and actional predictive signatures necessitates disruptive technological advances that will eventually change the practice of science and medicine. The scalability of these analysis pipelines and cost of conducting large-scale multiplex screening of patients in a clinical setting have room for overall improvement.

## 5 Regulatory standards

Regulations and guidelines concerning IHC-based IVDs vary among health authorities but generally become more stringent as the potential risk to the patient of a misdiagnosis increases. In the United States, these devices are regulated by the FDA, which classifies them as low (Class I), moderate (Class II), or high risk (Class III). Class III devices pose the greatest risk and typically require submission of an application for premarket approval as these are *de novo* devices without a similar preexisting marketed device. The PMA application^48^ includes non-clinical, clinical, hardware, software, and manufacturing modules that are submitted together or successively.

For FDA Class III devices, test results are preferably compared to an established truth, a so-called “gold standard,” with the intention that a measure of accuracy is linked to the clinical utility of the test. An accurate prediction of clinical response provides clinical utility data for both the drug and device.

Upon establishment of clinical relevance, a diagnostic might also be submitted without a PMA application for clearance in case of demonstration of equivalence or superiority to an approved marketed “predicate device” through submission of a 510(k) application^49^. In such instances, the risk is typically lower (Class II). The 510(k) *de novo* process has also been used by FDA to down-classify diagnostics that are considered “complementary” rather than “companion,” which would have previously been considered a higher-risk device.

IVDs used to identify new target analytes that potentially hold clinical significance are considered Class III if the diagnostic information thus obtained cannot be identified by conventional methods. For example, consider a cell phenotype that cannot be confirmed using a single IHC marker or approach, mBF or mIF, that provides more clinically useful treatment decisions than traditional single-marker chromogenic IHC.

Examples of lower-risk Class I IVDs are panels of single-marker IHC tests, used typically as an adjunctive to pathological diagnosis (Painter et al., 2010) to answer questions relevant to a specific clinical scenario such as anatomic, specimen or histologic type. Multiplex devices could reduce burden of testing. For example, undifferentiated solid tumors and heme tumors, such as lymphomas, require 10+ different markers to arrive at a proper diagnosis. While molecular profiling plays an important diagnostic role for these indications, there is greater confidence in pathological diagnosis when markers can be shown labeling different cell populations or cell/tumor compartments in the same slide.

### 5.1 AI/ML software as a medical device

In the United States, technologies such as scanners and software manufactured for digital pathology are considered medical devices and are regulated by the FDA. In 2017, the FDA approved the first digital pathology system for primary diagnosis using images of H&E-stained slides called the Philips IntelliSite Pathology Solution (Evans et al., 2018). Subsequently, other digital pathology companies have obtained 510(k) clearance for their devices. Due to the recent coronavirus disease 2019 (COVID-19) pandemic, the FDA and Centers for Medicare and Medicaid Services (CMS^50^) issued a guidance document to help expand the availability of devices and waivers to accommodate remote use. Originally purported as a temporary measure, the guidance/waiver potentially bodes well for digital pathology devices being submitted for evidence-based PMA filing or under section 510(k).

The COVID-19 pandemic is a likely catalyst for more pathologists to adopt a digital workflow (Kearney et al., 2021), as many realize the logistic and technical advantages of digital tools whose functionalities resemble conventional microscopy. For primary diagnosis, scanners are also meeting diagnostic image quality with spatial resolution that allows for the identification and recognition of key histological features (Lujan et al., 2021).

With regards to the regulatory evaluation of a digital WSI system, the FDA provides nascent guidance on the technical performance assessment data at the component level^51^. Component data may include scanner configuration such as light source and image optics, digital imaging sensor, image processing and composition software; workstation configuration such as image review and manipulation software, computer environment and display component. Such guidance ensures images intended for clinical uses are reasonably safe and effective for such purposes. Complementary white papers released by the Digital Pathology Association (DPA) also tackle the need for guidelines enabling safe adoption of IA in clinical practices (Aeffner et al., 2019; Lara et al., 2021).

Further, the FDA recently released a position paper on AI/ML systems that structure, integrate, and interpret large datasets to aid in clinical decision-making. The “Proposed Regulatory Framework for Modifications to AI/ML-Based Software as a Medical Device,”^52^ describes general principles regarding data management, retraining, and performance of Software as a Medical Device (SaMD) under Good Machine Learning Practices (GMLP). The prevalent model of this adoption requires the clinical pathologist to act as arbitrator for any AI/ML diagnostic decision, because the software makes no independent interpretations of the data. In this, anatomical pathology lags radiology where that field has pursued evidence to determine whether radiologist performance is the same, better, or worse if using computer-aided detection and/or computer-aided diagnostic interpretation of imaging findings (Zhu et al., 2022).

Digital Imaging and Communications in Medicine (DICOM^53^) is the standard for the communication and management of medical images and specifies how data quality is exchanged between medical imaging equipment and other systems whose operation is necessary for insight. Where whole-slide scanners previously produced files in their proprietary formats, DICOM Standards now provide support for WSI in pathology by incorporating a method of handling tiled large images as multi-frame images and multiple images of varying resolution (Herrmann et al., 2018; Badve et al., 2021). This approach allows pathologists to rapidly pan and zoom images at different magnifications. Annotations can be supported, and color consistency is defined using International Color Consortium^54^ profiles.

As expected, significant differences in data characteristics of histopathological slides scanned with different WSI systems become evident, which pose a particular issue for AI/ML when the algorithm derived with a training set or supervised model of learning does not perform well on real-world unseen or unsupervised data sets. Several international computational pathology challenges (e.g., ILSVRC^55^, TUPAC^56^, CAMELYON^57^, ACDC-LungHP^58^, KPMP^59^) using public datasets are addressing such issues. Beyond sample heterogeneity, “domain shift challenges” have been ascribed to scanner properties such as color balance/profile, lighting intensity/brightness and optical contrast variations or aberrations (Kohlberger et al., 2019).

#### 5.1.1 Clinical decision points—Software validation

Given that the unique features of SaMD may extend regulatory frameworks beyond a traditional medical device, guidance supporting innovation and access to SaMD globally is being supported through the International Medical Device Regulators Forum (IMDRF^60^), of which the FDA is one member and Chair of a Working Group established in 2013. Particularly, this Forum guides “the assessment and analysis of a SaMD’s clinical safety, effectiveness and performance as intended by the manufacturer in the SaMD’s definition statement.”

Clinical validation is necessary for any SaMD (Fraggetta et al., 2021) as determined by the manufacturer before (pre-market) and after (post-market) distribution to establish a relationship between verification and validation results of an algorithm and the clinical conditions of interest (Carolan et al., 2022). Prior to routine use, it is important to evaluate solutions that automatically extract information from digital histology images, and their predictive performance (Homeyer et al., 2022). Various other technical and business challenges must similarly be overcome to commercialize digital pathology solutions (Kearney et al., 2021; Lujan et al., 2021). Despite extensive research developments, few SaMDs have such potential. Prototypic software is often coded quickly without quality considerations, thereby making them prone to runtime errors and difficult to maintain, vulnerable to security holes and unlikely to enable feature extensions (Zinchenko et al., 2022).

Moving toward an all-digital healthcare solution presents its own unique challenges, namely patient confidentiality. Introduced in 1996, HIPAA provides privacy and security for protected health information. In Europe, the new General Data Protection Regulation (GDPR^61^) provides EU citizens a “right to be forgotten” if a request is received to remove an individual’s personal information from a data set used for scientific research - regardless of whether the individual previously provided consent for use of their data. The recent Digital Health Innovation Action Plan^62^ is a notable shift in the FDAs approach to digital health technologies, and an important step in FDA regulation of this area. Even if data storage and image management systems are not considered part of a medical device (and therefore not regulated by the FDA), they will likely be subject to HIPAA compliance, safety, and quality standards.

## 6 Safety and quality standards

From a safety perspective, a CDx may identify patients who respond positively or negatively to a therapy, which can be factored into treatment decisions. In the drug development process, a clinical trial’s potential success can be improved by using a biomarker for patient selection and stratification. If patients with the biomarker are more likely to respond, the number of positive outcomes may be increased, and the size and cost of the study reduced due to fewer patients in the trial.

Quality standards are included at all stages of diagnostic development, from validation to inline quality monitoring and proficiency testing. Manufacturing and quality control processes for the reagents and other assay components are subjected to similar development and/or stability studies as the IVD CDx assay itself, including optimization testing. These studies’ outputs feed directly into the draft specifications tested in the project’s subsequent verification and validation. For quantitative tests—or qualitative tests with a quantitative underpinning—linearity studies are also expected to ensure the test reports consistently across an expected range.

### 6.1 Analytic standardization in IHC

One critical and historical problem for IHC has been the lack of reference standards or calibrators, at least as defined by World Health Organization (WHO^63^), National Institute of Standards and Technology (NIST^64^) or other international standards organizations such as the Bureau International des Poids et Measures (BIPM^65^).

NIST has recently worked with a few academic centers and reagent manufacturers on a single-target basis for known predictive biomarkers (e.g., PD-L1, ER, p53—Atha et al., 2010; Torlakovic et al., 2021; Sompuram et al., 2022) for the creation of tools linked to international standards enabling IHC measurement traceability and their validation by IPT/EQA programs. A consensus call supported by CAP, NIST, and FDA to develop a HER2 standard reference material occurred prior but no test standard emerged (Hammond et al., 2003).

#### 6.1.1 Clinical decision points—Reference standards

Reference standards are used to establish upper and lower limits of quantification (i.e., ULOQ, LLOQ) and working/reportable ranges (i.e., linearity) to establish assay analytical sensitivity—LOD should not change when new reagent lots are used in the test, since LOD typically represents the threshold separating a positive result for an analyte from one that is negative.

The inability to generate analytic response curves for commonly used clinical IHC tests in part or whole complicates the standardization of test results, because calibrators are used to establish variability between and within laboratories, and for operational qualification of new lots of assay components.

With mIHC, there is minimal standardization of antigen- or reporter-specific calibrators or batch-run controls. Like the subsequent steps that comprise the analytic phase of the test, proper antigen retrieval is not usually verified with the benefit of an external control; distinguishing between staining variability caused by antigen retrieval (preanalytical) or alterations in staining reagents or procedure (analytic variability) is an identified area that needs addressed for clinical deployment of mIHC. Efforts are underway to develop and validate batch run controls using cell pellets and/or curated tissue microarrays. Nonetheless, these approaches have been more suited to visual review (descriptive or qualitative, Yes/No, or Positive/Negative readouts—Torlakovic et al., 2014; Torlakovic et al., 2015; Eisen, 2017) than quantitative non-optical methods such as IA.

For mBF, spectral absorption features of chromogens, especially co-localized, typically prevent reliable per-target quantitation when using a typical red-green-blue (RGB) sensor found in most microscope cameras. RGB sensors lack the spectral resolution to accurately unmix, or de-convolute component spectra, for independent quantitation. In recent years, new BF colors with chemistry based on FL dyes have become available that form new colors when combined (Morrison et al., 2020). The use of these, which are translucent and complementary colors opposite in the visible spectrum, provide clearer visualization of co-stained cell populations and facilitate more robust automated image analysis and processing by different AI-based software.

For mIF, quantitation is based on isolating marker fluorophore emissions from one another, either by purely optical means, or by implementing spectral unmixing techniques which is needed for accurate quantitation of more than four co-localized markers. In such circumstances, spectral unmixing compensates for channel crosstalk and support accurate quantitation, like detector spillover compensation methods in flow cytometry, but performed on a per-pixel basis.

When using FFPE for mIF, MSI also isolates marker fluorophore emissions from AF. Unmixing spectral libraries are developed using control tissues stained with one fluorophore at a time, including an unstained slide to provide an AF spectrum.

Multispectral imaging is the first step toward reliable compensation-based quantitative measurement of mIF and, more broadly, significantly improves the ability of mIF assays to be effectively quantified by IA platforms since the overall signals within a given pixel can be partitioned correctly into its different FL components. MSI alleviates the major challenge associated with IF and FFPE samples: reduction of the impact of AF, which is particularly important for fluorophores at the blue/green end of the visible spectrum (Mansfield et al., 2008; Prost et al., 2016). MSI can also be applied to mBF (Levenson, 2006; van der Loos, 2008; Morrison et al., 2020). That said, AF within the sample is not physically eliminated, and noise may continue to compromise results especially for dim signals of low abundance targets.

#### 6.1.2 Clinical decision points—Antibody specificity

Another challenge to standardization in IHC is the so-called “reproducibility crisis”^66^ in preclinical research, with antibody specificity identified as the central issue (Baker, 2015). Under 21 CFR 820, a critical output from the CDx design validation process is the ability to test several GMP lots of final antibody product and establish acceptable lot-to-lot performance. While such material can pass manufacturing quality checks, if analytical specificity has not been rigorously defined, then these ASRs and final IVD CDx devices will deliver poor performance in the field where such performance variables are critically necessary.

Experiences documented by Nordic Immunohistochemical Quality Control (NordiQC^67^), one of several IPT/EQA programs established to evaluate inter-laboratory consistency, have described approximately 30% of diagnostic tests in their general module—tests for the most common epitopes demonstrated in surgical and clinical pathology to identify and subclassify neoplasms—as insufficient or inappropriate for use (Nielsen, 2015). Similar double-digit failure rates have been reported by other IPT/EQA programs, with mostly false-negative test results.

Unfortunately, such national programs or umbrella consortiums (e.g., UK NEQAS ICC^68^, cIQc^69^, RCPAQAP^70^, IQN Path^71^) are focused on the examination of BF IHC rather than IF. Although each uses different approaches in evaluating the performance of individual participating laboratories, all strive to achieve consistency and accuracy in the operation of clinical laboratories with the goal of improved patient safety.

## 7 Diagnostic approval routes

The ability to identify a biomarker’s predictive nature early in a therapeutic’s development is not always straightforward, as pointed out within the FDAs own enrichment strategy guidance. Often, the relationship to drug response is identified retrospectively, and additional trials must be conducted to confirm results.

Traditional IVDs detect one or a small number of analytes and diagnose one or a small set of conditions. mIHC-based diagnostics, by virtue of assay complexity, do not easily fit a proven model of IVD test development.

Recently introduced by the FDA, expedited access programs (EAP^72^) that accelerate approval and enable priority review designation (e.g., a Breakthrough Device Pathway, part of the Pre-submission process) for products targeting an unmet medical need, treating a serious or life-threatening condition, and providing clinically meaningful advantages over other on-market devices. Despite its compelling attribute, the accelerated approval scenario would likely drive the first clinical application of mIF and will present challenges of validation and verification of mIF, given some operator dependencies. Such dependencies may include pathologist input, oversight, or the involvement of software-assisted interpretation.

Traditional diagnostic approval routes require the revalidation of each change made in a mIHC IVD CDx assay, with each a step in a linear process that could require skilled operator involvement and intervention that could potentially impact the final test result. This is especially true when the pathologist’s involvement includes tissue quality assessment (usually in concert with H&E), tumor annotation or FOV selection, and results review and sign-off.

### 7.1 LDT

Laboratory Developed Tests, LDTs, have been a cost-effective way to bring forward complex technology or multiparameter data analysis needs into the diagnostic space, with regulatory oversight provided by CLIA and CAP rather than the FDA. LDTs analytically validated to be CLIA-certified and/or in compliance with CAP-guidelines are typically viewed as necessary for inclusion in drug trials to support primary or secondary endpoints, and to provide reliable data to support consideration for moving a test toward patient selection and eventual CDx use.

FDA officially holds discretionary authority to regulate LDTs under the Public Health Service Act, and has published guidelines^73^, but has yet to exercise this authority so as to not disrupt existing practice that has been serving patients effectively for many years. In the past, the extent of the FDA’s jurisdiction in this area caused disagreement, particularly as tests are not physically distributed or delivered outside the originating laboratory. As a workaround, the FDA exercised some control over LDTs by limiting claims on ASRs (Caliendo and Hanson, 2016), which are subject to specific FDA requirements, including their sale and labeling. CAP, which accredits many clinical laboratories under CLIA, took the middle ground in the argument by proposing^74^ the FDA should only review LDTs that are at the very highest risk level (Class III).

ASRs are key components of LDTs and some other diagnostic tests, subject to GMP and defined under 21 CFR 864^75^ as “antibodies, both polyclonal and monoclonal, specific receptor proteins, ligands, nucleic acid sequences, and similar reagents which, through specific binding or chemical reaction with substances in a sample, are intended for use in a diagnostic application for identification and quantification of an individual chemical substance or ligand in biological specimens.”

LDTs, as a locked device or platform, are comprised of a set of reagents (e.g., ASRs), sample preparation, instrumentation, software, and test scoring/interpretation guidelines that are validated together. The laboratory provider for clinical trial testing acts as an LDT manufacturer. Traditional IVD CDx are developed by a conventional IHC diagnostic manufacturer (e.g., Agilent/Dako^76^, Roche/Ventana^77^ or Danaher/Leica^78^ in the IHC space). As such, the test provider is dependent on external suppliers for the critical raw materials used in the manufacture of the LDT.

Importantly, since an LDT provider is typically not the manufacturer of the equipment used to run the final assay, the assay remaining in a validated state through changes to the hardware or software that are outside the control of the LDT manufacturer becomes difficult to assure. This point is particularly salient for machines such as autostainers that run contemporary multiplexing methods, i.e., those offering some of the highest levels of antibody multiplexing (ultrahigh-plex, 10+ markers). Simultaneously, these machines are also burdened by high cost, maintenance complexity, and low throughput.

#### 7.1.1 ImmunoPROFILE

A recent example of an LDT where spatially resolved technology has been included in clinical decision-making at the local level is PROFILE^79^, a large-scale cohort research study jointly by Dana-Farber/Brigham and Women’s Cancer Center (DF/BWCC) and Dana-Farber/Boston Children’s Cancer and Blood Disorders Center (DF/BC). A mIF LDT workflow (ImmunoPROFILE) based on the PhenoImager HT platform was developed and extensively validated on 1,000+ patient samples and 16+ tumor types to provide a spatial and quantitative assessment of tumor and immune cells within the TME for all immunotherapy-eligible patients. Results are ordered by and sent back to a physician and are used as an aid to learn about patient eligibility for current clinical trials.

#### 7.1.2 Multianalyte assays with algorithmic analyses—ProMark and TissueCypher

Several other LDTs that are multianalyte assays with algorithmic analyses (MAAAs) incorporate results from a panel of tests, with or without other clinical information, into an algorithm to generate a risk or probability score. These have diagnostic superiority in diseases in which single biomarkers have limited validity or a non-invasive biomarker is lacking.

ProMark^80^ is a biopsy-based prognostic test that utilizes mIF (eight protein biomarkers) to evaluate FFPE prostate tissue to differentiate indolent from aggressive prostate cancer, and to generate an algorithmically derived risk score indicating the likelihood of having high-risk disease.

TissueCypher^81^ is a diagnostic test that predicts the risk of developing high grade dysplasia or esophageal cancer in patients with Barrett’s Esophagus, as an aid to pathologist interpretation of standard pinch biopsies from upper GI endoscopy procedures. This combines quantitative analysis of nine protein biomarkers with tissue structure information, reported as an individualized risk score to help prescribers and patients understand risk of progression to high grade dysplasia or cancer.

### 7.2 PMA

LDTs can only be run in a single laboratory which poses a significant restriction to the global testing footprint and makes LDTs less appealing for biopharma because of its impedance to drug adoption and use. Excitingly, the current trend for IO is that Phase IIb are being used as pivotal or registrational trials, and thus smaller regional studies are more frequently including patient pre-qualification that would benefit from novel technology use.

Single-site PMAs (ssPMA) offer a practical interim step between LDT CDx and distributed IVD CDx, when the assays depend on new and complex methods and technology, such as with mIF. ssPMAs are essentially LDTs that are cleared through the FDA process, which requires analytical validation aligned with standard PMAs for distributed IVDs and Class III medical devices but reduced in scope because the test is run at only one site.

As with distributed IVD CDx, ssPMAs require post-market surveillance of assay performance (pharmacovigilance, Phase IV) and a proactive and systematic risk-benefit process that gathers and analyzes quality, performance, and safety data; few non-IVD manufacturers have experienced this. Concomitantly, only a few LDT PMAs have been filed as IVDs and cleared for approval by the FDA.

### 7.3 Strategic considerations

LDTs are an attractive strategy for fast market entry *via* the PMA process utilizing centralized testing at a single site and, as such, often include the use of highly complex technologies or technological advances. Nevertheless, ssPMA may also follow EAP special designation for approval if meeting unmet medical needs or providing clinically meaningful advantages over other on-market devices.

Compared with the PMA process in the United States, the EU self-certification IVD procedure (CE-IVD^82^) that complies with the European *In Vitro* Diagnostic Devices Directive 98/79/EC (IVDD^83^) has a different regulatory path, one that has recently been strengthened by the new *In Vitro* Diagnostic Regulation 2017/746 (IVDR^84^) to align with current perceptions about the role and approval of CDx assays by the FDA.

However, under IVDR clinical laboratories running LDTs on EU patient samples will be significant impacted, regardless of whether that laboratory is in the European Union or elsewhere. From 2022, LDTs under IVDR will require CE-IVD marking. No “grandfathering” of existing LDTs will be allowed. Unless developers of existing LDTs can betterment than equivalent CE-marked tests, with appropriate clinical evidence to support their intended purpose, these LDTs must be removed from the market.

IVDR also requires that medical devices such as digital pathology software and slide scanners have CE-markings and ensure that all digital platforms in or entering the market are thoroughly validated. Scanners and associated software of numerous manufacturers (e.g., Hamamatsu, 3DHistech, Aperio, Philips) are currently CE-IVD labeled under IVDD. Under IVDR and resembling current FDA approval standards, a performance evaluation will be required, including a scientific validity report and analytical and clinical performance data. To date, only two systems - the Philips IntelliSite Digital Pathology Solution (Evans et al., 2018) and Leica Biosystem’s Scanner AT DX/Sectra DP Module (Bauer et al., 2020) - have been granted FDA approval for primary histopathological diagnosis.

#### 7.3.1 Clinical decision points—VALID and VITAL acts

Legislation introduced in the United States Congress in 2020, the Verifying Accurate, Leading-edge IVCT Development (VALID^85^) Act, enacted attempts to bring about FDA regulation of IVD test kits and LDTs under the same umbrella, with a new type of medical device: the *In Vitro* Clinical Test (IVCT). The VALID Act proposes a risk-based framework for IVCT regulation. High-risk novel tests require approval requirements comparable to existing medical device regulations, while lower and moderate-risk tests might go to market after passing through technological certifications. Unlike IVDR, the VALID Act would grandfather existing LDTs in clinical use (Graden et al., 2021).

VALID would also give the FDA ability to regulate LDTs, where previously the Agency had not exercised enforcement discretion over LDTs, thereby leaving CMS to oversee these under CLIA. Another bill recently introduced, the Verified Innovative Testing in American Laboratories (VITAL^86^) Act, seeks again to redress the balance to exclude FDA from any oversight role over LDTs and place them solely under the oversight of CMS/CLIA regulations.

Beyond the United States and EU, the regulatory landscape around the globe is equally complex. Regulations pertaining to IVD products continue to evolve, although they are becoming more harmonized with international standards.

## 8 Conclusion

CDx tests typically work by identifying a biomarker’s attributes (presence, absence, or amount associated with a therapy’s response) or by assessing physiological or anatomical patient characteristics that might affect response.

Maturing as an analytical platform, mIF provides reliable and reproducible results that may be generated in a clinically suitable, high-throughput workflow. The spatial biology approach provides several advantages over other biomarker modalities by enabling deeper interrogation of the intricate cellular- and protein-level co-expression, localization, and arrangement within the TME.

**FIGURE 6:**
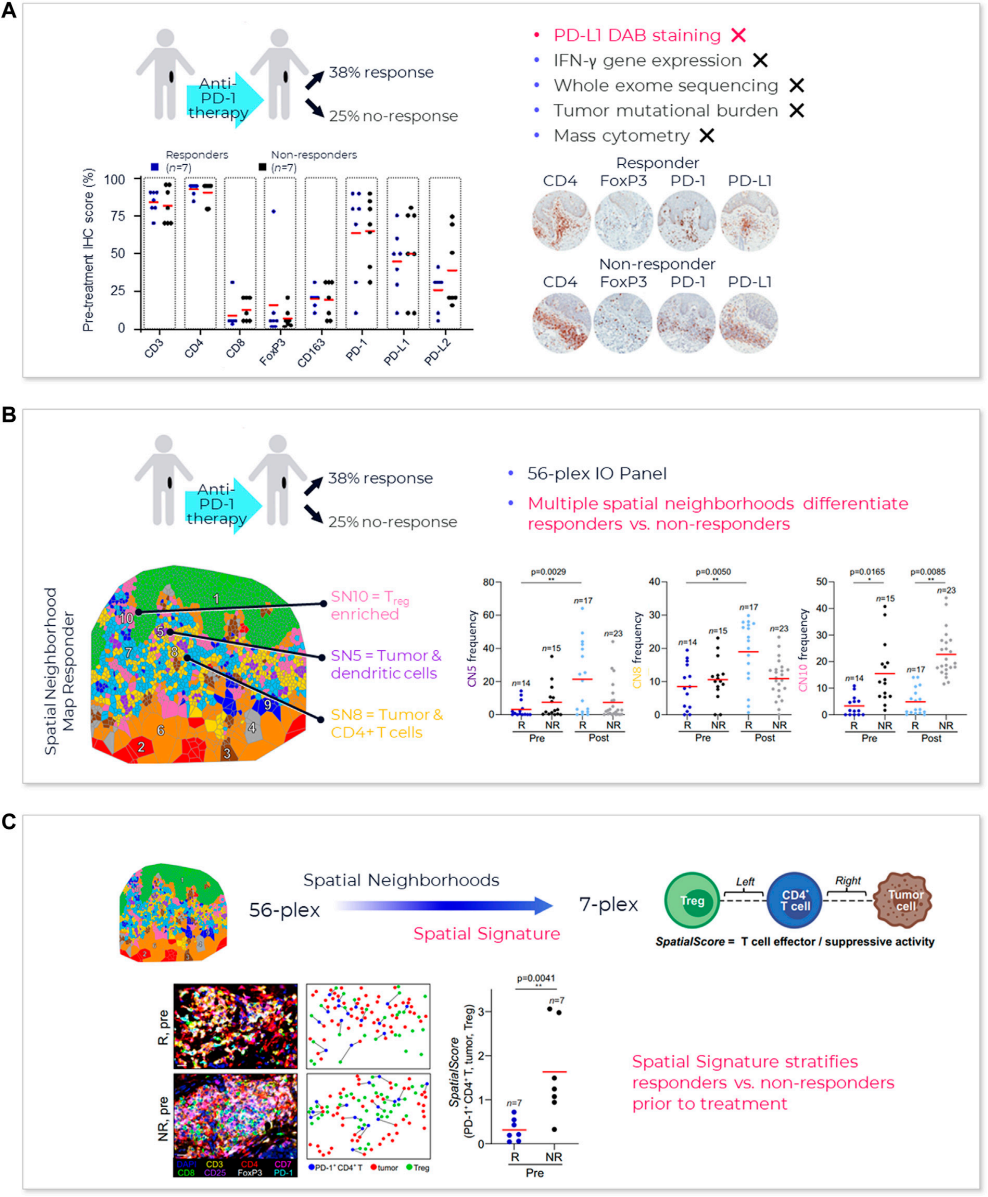
**mIF better predicts patient response—lymphoma**. **(A)** Conventional biomarkers fail to predict outcomes in lymphoma (Phillips et al., 2021). **(B)** Spatial neighborhood analysis identified differences in TME spatial organization in responders vs. non-responders - cellular neighborhoods (CNs) enriched in tumor and dendritic cells (CN-5, purple region, *p* = 0.0029) and tumor and CD4^+^ T cells (CN-8, yellow region, *p* = 0.005) were present at significantly higher frequencies in responders’ post-treatment compared to other groups suggesting a more immune-activated TME observed in responders following pembrolizumab therapy. Treg enriched CN (CN-10, pink region, *p* = 0.0165 and 0.0085) was present at a significantly higher frequency in non-responders than responders pre- and post-treatment (Phillips et al., 2021). **(C)** High-plex analysis identified three key cellular neighborhoods with differences in responders vs. non-responders. This data was then focused using the PhenoImager HT to enable greater throughput and direct investigation of the potential utility of a SPS in clinical samples. A lower SPS (CD4+ T cells are closer to tumor cells than Tregs) suggested increased T cell effector activity and higher SPS (iCD4+ T cells are closer to Tregs than tumor cells) suggested increased T cell suppression. Distances between a specific tumor and immune cell types could be employed to identify a predictive biomarker of immunotherapy response (CD4+ cells, tumor cells, and Tregs) (Phillips et al., 2021). Publication copyright for Phillips et al., 2021 is governed by a CC BY 4.0 License.

**FIGURE 7:**
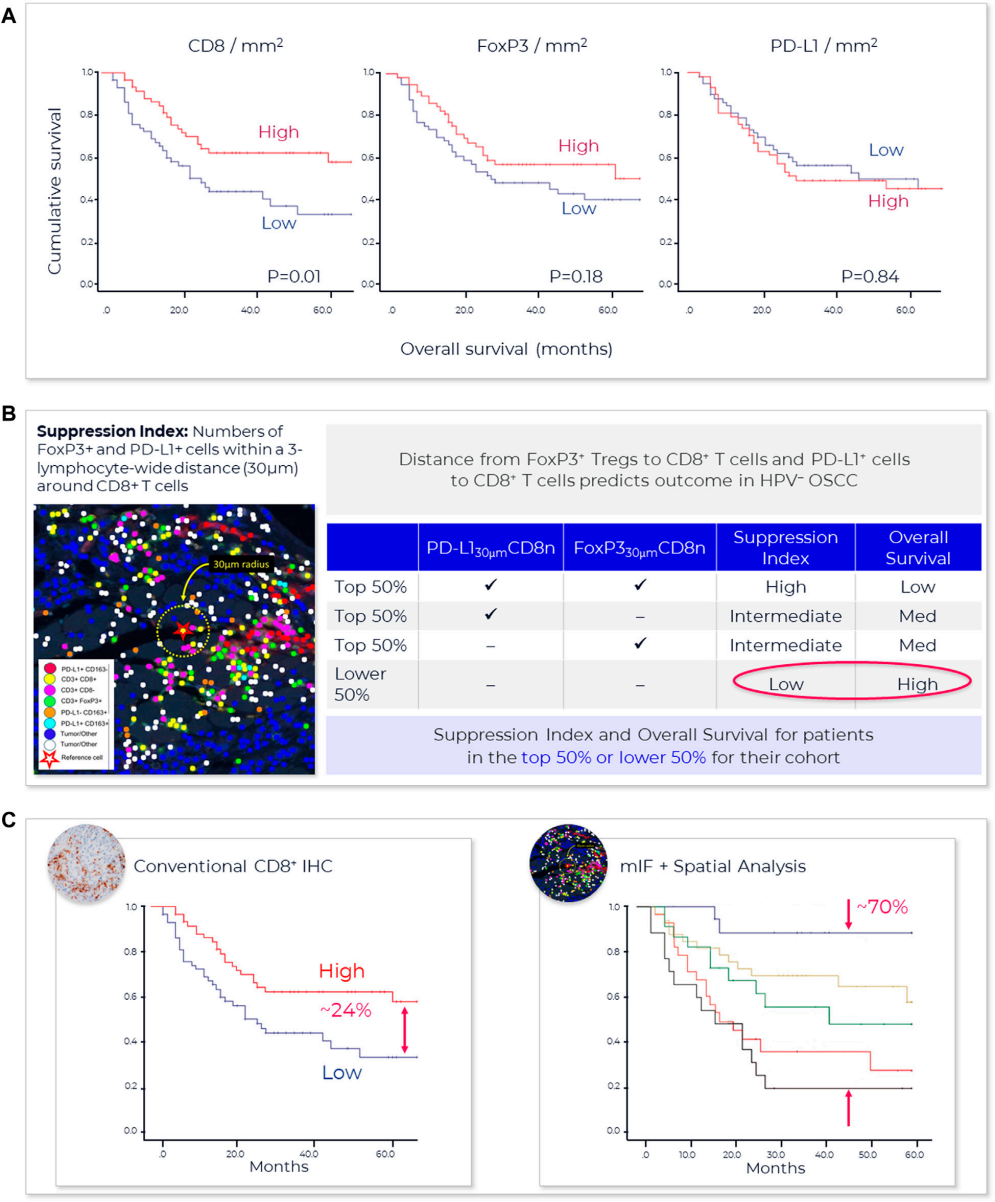
**mIF better predicts patient response—HNSCC**. **(A)** Immune cell infiltrate densities at the tumor invasive margin are insufficient to predict favorable overall survival (OS) for oral squamous cell cancer patients. CD8+, FoxP3+, and PD-L1+ cell densities on both the tumor and stromal sides of the invasive margin were examined. Although higher densities CD8+ T cell showed some prognostic value, sufficient classification was lacking (Feng et al., 2017). **(B)** A positive correlation between an increased number of Tregs (FoxP3+) and CD8+ T cell infiltrates was observed; authors postulated that Tregs might not be close enough to the CD8+ T cells to suppress their effector function. In response, they developed a “Suppression Index.” This index reflected the number of FoxP3+ and PD-L1+ cells within a 3 “lymphocyte wide” or a 30 μm distance around CD8+ T cells. Using this index, patients were ranked for number of PD-L1+ and FoxP3+ cells within 30 μm distance around CD8+ T cells. Patients who were ranked in the top 50% for both PD-L1+ and FoxP3+ cells had a high suppression index with a low overall survival while those that did not rank in the top 50% for either PD-L1+ and FoxP3+ cells had a low suppression index and a high overall survival rate (Feng et al., 2017). **(C)** Analysis of the entire cohort demonstrates that by combining the suppressive index of both the stromal and tumor side of the invasive margin provides a cumulative suppressive index scoring system that better stratifies patients than conventional IHC. Multiplex IF plus spatial analysis of the proximity of FoxP3+ and PD-L1+ to CD8+ cells lead to a highly significant stepwise reduction of overall survival based on an increasing cumulative suppressive index and the development of a highly indicative prognostic marker superior to the prognostic index of the single markers (Feng et al., 2017). Publication copyright for Feng et al., 2017 is governed by a CC BY 4.0 License.

Increasingly sophisticated data analysis tools have facilitated the identification of a subset or subsets of biomarkers using a SPS which can predict a disease state, determine the likelihood of disease progression, or calculate the probability of responding to therapy (Figures 6, 7). Multiplex IHC is driving, and being driven by, this revolution in IA and AI/ML to support more complex assessments of expression levels, cell positivity and show that spatial context matters.

Immune checkpoint inhibitor therapy has clearly demonstrated efficacy for subsets of late-stage patients, and the range of therapeutic options is rapidly expanding, with greater than 80 FDA approvals involving anti-PD-1/PD-L1 antibodies since 2014^87^, typically combined with other ICIs or conventional therapies. With such a broad range of options, need for better predictive tests is driven by relatively low response rates, sometimes significant side effects, and very high treatment costs, which essentially double when two ICIs are combined. mIF CDx tests have the potential to add value to the drug development process by identifying patients who are more likely to respond to a particular therapy or combination of therapies, thereby improving treatment outcomes.

For example, a choice between administering anti-PD-1/PD-L1 monotherapy, which can be very effective by itself, and combining it with other ICIs (e.g., CTLA4, Lag-3) or chemotherapy has material implications for the patient. Albeit a genetic test, Exact Science’s Oncotype Dx^88^ plays a role for ER+ breast cancer patients receiving endocrine therapy and provides specific information on disease recurrence (prognostic value) and if there is a benefit to chemotherapy or radiation therapy (predictive value). Similar needs exist today with many of the anti-PD-1/PD-L1 eligible patients, in particular patients with late-stage NSCLC and Head and Neck (H&N) cancers.

An emerging need for mIHC is in the neoadjuvant and adjuvant settings, where trials are showing promise but: patient populations are substantially larger than in the metastatic setting, surgery alone often can cure, and the health system is not capable of supporting broad application of these expensive therapies. Cases in point are recent trial results from intralesional injection of ICIs, for ductal carcinoma *in situ* (DCIS) and triple negative breast cancer^89^.

Despite many advantages, substantial workflow and infrastructure challenges still act as barriers to adoption that will need to be addressed before mIHC (mBF and mIF) can fully enter clinical practice. That said, while spatial biology involving mIF has mostly been applied toward research applications to date and early-phase exploratory trials, there are signs of progress towards clinical utility or later stage clinical trial use (Table 3). Approval of a multiplex solution will require co-developing the test alongside the therapeutic, and demonstrating benefit, in partnership with a diagnostic manufacturer with appropriate mIHC capability/ies^90^.

**TABLE 3:** Clinical trials utilizing multiplex immunohistochemistry and other spatial imaging technologies. Adoption of spatially resolved multiplexing technologies in pivotal clinical trials, suggesting a role for spatial signatures as both prognostic and predictive indicators for therapeutic benefit^92^. Over the next 3–5 years, transition from small exploratory studies to large scale registrational studies is anticipated.

Trial id	Responsible parties	Tech provider	Phase/Trial type	Description
NCT01042379^a^	QuantumLeap Healthcare Collaborative, University of California San Francisco	Akoya	Ph2, Retrospective	Neoadjuvant and Personalized Adaptive Novel Agents to Treat Breast Cancer (I-SPY: Investigation of Serial Studies to Predict Your Therapeutic Response With Imaging And moLecular Analysis 2)
NCT02785250^b^	IMV Inc	Akoya	Ph1b/2, Retrospective	Study of DPX-Survivac Therapy in Patients With Recurrent Ovarian Cancer
NCT04688658^c^	Bristol Myers-Squibb, Secura Bio Inc	Akoya	Ph1/2, Prospective	Duvelisib in Combination With Nivolumab in Patients With Advanced Unresectable Melanoma
NCT04963283^d^	National Cancer Institute, Criterium Inc., Bristol Myers-Squibb, Exelixis	Akoya	Ph2, Prospective	Study of Cabozantinib and Nivolumab in Refractory Metastatic Microsatellite Stable (MSS) Colorectal Cancer
NCT00114816^e^	Finnish Breast Cancer Group, Hoffmann-La Roche, Sanofi, AstraZeneca	Nanostring	Ph3, Retrospective	Docetaxel Followed by CEF (Cyclophosphamide, Epirubicin and 5-Fluorouracil) Compared to Docetaxel and Capecitabine Followed by CEX (Cyclophosphamide, Epirubicin and Capecitabine) as Adjuvant Treatment for Breast Cancer (FinXX)
NCT04895761^f^	Providence Health and Services	Nanostring	Ph1b, Prospective	Neoadjuvant DPX-Survivac Aromatase Inhibition, Radiotherapy or Cyclophosphamide in HR+ HER2- Breast Cancer
NCT04622423^g^	IRCCS San Raffaele, Università Vita-Salute San Raffaele	10X Genomics	Ph?, Prospective	Advanced Therapies for Liver Metastases (LiMeT)
NCT05371756^h^	Baylor Research Institute	10X Genomics	Ph?, Prospective	Collection of Patients’ Biospecimens for Analysis of Immunological and Molecular Biomarkers (TIOB)
NCT03299946^i^	Sidney Kimmel Comprehensive Cancer Center at Johns Hopkins, Bristol Myers-Squibb, Exelixis	Standard BioTools	Ph1b, Retrospective	Feasibility and Efficacy of Neoadjuvant Cabozantinib Plus Nivolumab (CaboNivo) Followed by Definitive Resection for Patients With Locally Advanced Hepatocellular Carcinoma (HCC)
NCT03669601^j^	Cambridge University Hospitals NHS Foundation Trust, AstraZeneca	Standard BioTools	Ph1, Retrospective	AZD6738 and Gemcitabine as Combination Therapy (ATRiUM)
NCT04009967^k^	CHU de Quebec-Universite Laval, Merck Sharp & Dohme LLC.	Standard BioTools	Ph2, Prospective	Biomarkers for Neoadjuvant Pembrolizumab in Non-Metastatic Prostate Cancer Positive by 18FDG-PET Scanning (PICT-01)
NCT04393285^l^	Gynecologic Oncology Group Foundation, Eli Lilly and Company	Standard BioTools	Ph2, Prospective	Abemaciclib and Letrozole to Treat Endometrial Cancer
NCT04951154^m^	Janssen Research and Development LLC.	Standard BioTools	Ph?, Prospective	A Study to Examine Biomarkers From Lung and Blood Samples in Participants With Suspected Lung Cancer
NCT04053673^n^	Ribon Therapeutics Inc	IonPath	Ph1, Retrospective	Phase 1 Study of RBN-2397, an Oral PARP7 Inhibitor, in Patients With Solid Tumors
NCT04068194^o^	National Cancer Institute	IonPath	Ph1/2, Prospective	Testing the Combination of New Anti-cancer Drug Peposertib With Avelumab and Radiation Therapy for Advanced/Metastatic Solid Tumors and Hepatobiliary Malignancies
NCT02528357^p^	GlaxoSmithKline, Merck Sharp & Dohme LLC.	Leica	Ph1, Retrospective	GSK3174998 Alone and With Pembrolizumab in Participants With Advanced Solid Tumors (ENGAGE-1)
NCT03506373^q^	National Cancer Institute, Mayo Clinic	Leica	Ph2, Prospective	Ibrutinib and Ixazomib Citrate in Treating Patients With Newly Diagnosed, Relapsed or Refractory Waldenstrom Macroglobulinemia
NCT05163041^r^	BicycleTx Limited	Leica	Ph1/2, Prospective	Study BT7480-100 in Patients With Advanced Malignancies Associated With Nectin-4 Expression
NCT03291002^s^	CureVac, Syneos Health, Cromos Pharma LLC.	Nanostring/Leica	Ph1, Retrospective	Study of Intratumoral CV8102 in cMEL, cSCC, hnSCC, and ACC

Assumptions and Notes

^a^ https://clinicaltrials.gov/ct2/show/NCT01042379

^b^ https://clinicaltrials.gov/ct2/show/NCT02785250

^c^ https://clinicaltrials.gov/ct2/show/NCT04688658

^d^ https://clinicaltrials.gov/ct2/show/NCT04963283

^e^ https://clinicaltrials.gov/ct2/show/NCT00114816

^f^ https://clinicaltrials.gov/ct2/show/NCT04895761

^g^ https://clinicaltrials.gov/ct2/show/NCT04622423

^h^ https://clinicaltrials.gov/ct2/show/NCT05371756

^i^ https://clinicaltrials.gov/ct2/show/NCT03299946

^j^ https://clinicaltrials.gov/ct2/show/NCT03669601

^k^ https://clinicaltrials.gov/ct2/show/NCT04009967

^l^ https://clinicaltrials.gov/ct2/show/NCT04393285

^m^ https://clinicaltrials.gov/ct2/show/NCT04951154

^n^ https://clinicaltrials.gov/ct2/show/NCT04053673

^o^ https://clinicaltrials.gov/ct2/show/NCT04068194

^p^ https://clinicaltrials.gov/ct2/show/NCT02528357

^q^ https://clinicaltrials.gov/ct2/show/NCT03506373

^r^ https://clinicaltrials.gov/ct2/show/NCT05163041

^s^ https://clinicaltrials.gov/ct2/show/NCT03291002

One example of this is the OncoSignature™ mIF test under development by Akoya in collaboration with Acrivon Therapeutics^91^. This test is being used^92^ for patient enrollment in an ongoing clinical trial (https://clinicaltrials.gov/ct2/show/NCT05548296) and, if it accurately establishes therapy benefit, will be an FDA-approved IVD for a kinase inhibitor of DNA damage response.

**FIGURE 8:**
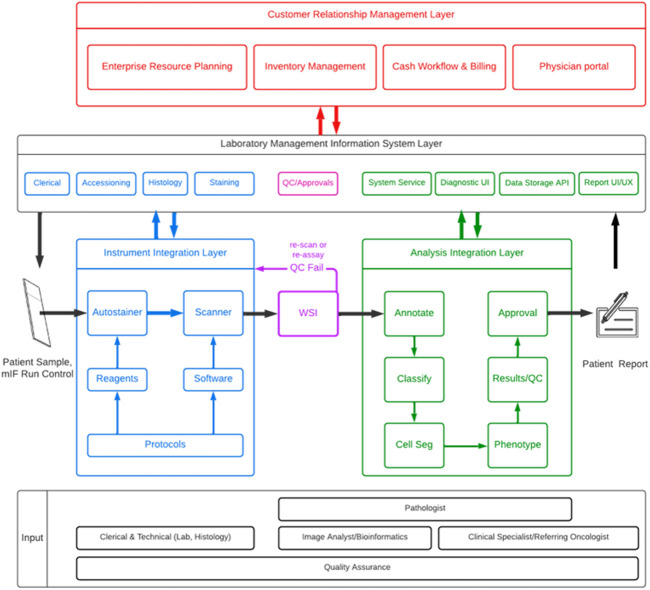
**Example order-to-result mIF workflow**. This schematic represents one example of a complete deployable mIF lab process, showing the multiplicity of steps inherent within the process from sample-to-result and some of the factors that can impact the outcome that might be considered outside the mIF technical process. Lab workflow comprises a series of individual layers/solutions that respect and exploit the unique characteristics and needs for each operation; no two labs are the same, so specific workload processes and loads may be different, and components of this end-to-end process may also be facility-specific (e.g., autostainer and scanner, digital pathology software, overarching LIMs and CRM).

The prevalent model of technology adoption as well as the ability to integrate treatment information from mIF will determine the future relationship of this modality with that of the clinical pathologist (Figure 8). Nevertheless, the demand for a better understanding of the TME, the tumor and its interaction with different cell phenotypes of the immune system, perhaps as defined by SPS, provides the impetus to overcome any limitations.

## Footnotes

1
https://seer.cancer.gov/

2
https://fnih.org/our-programs/partnership-accelerating-cancer-therapies-pact

3
https://www.sitcancer.org/pathology/mxif

4
https://www.fda.gov/

5
https://www.ema.europa.eu/en

6
https://www.ich.org/

7
https://www.fda.gov/medical-devices/premarket-submissions-selecting-and-preparing-correct-submission/investigational-device-exemption-ide

8
https://www.cdc.gov/phlp/publications/topic/hipaa.html

9
https://www.fda.gov/regulatory-information/search-fda-guidance-documents/distribution-in-vitro-diagnostic-products-labeled-research-use-only-or-investigational-use-only

10
https://www.cdc.gov/clia/index.html

11
https://www.cap.org/

12
https://clsi.org/

13
https://www.iso.org/home.html

14
https://www.iso.org/standard/56115.html

15
https://www.wadsworth.org/regulatory/clep

16
https://www.fda.gov/medical-devices/premarket-submissions-selecting-and-preparing-correct-submission/premarket-approval-pma

17
https://nordiqc.org/downloads/documents/137.pdf

18
https://www.ema.europa.eu/en/ich-q2r2-validation-analytical-procedures

19
https://www.cap.org/protocols-and-guidelines/cap-guidelines/current-cap-guidelines/principles-of-analytic-validation-of-immunohistochemical-assays

20
https://www.fda.gov/medical-devices/guidance-documents-medical-devices-and-radiation-emitting-products/guidance-submission-immunohistochemistry-applications-food-and-drug-administration-final-guidance

21
https://clsi.org/standards/products/immunology-and-ligand-assay/documents/i-la28/

22
https://seer.cancer.gov/

23
https://www.cancer.net/

24
https://www.fda.gov/drugs/types-applications/investigational-new-drug-ind-application

25
https://www.fda.gov/regulatory-information/search-fda-guidance-documents/requests-feedback-and-meetings-medical-device-submissions-q-submission-program

26
https://www.fda.gov/about-fda/fda-organization/center-drug-evaluation-and-research-cder

27
https://www.fda.gov/about-fda/fda-organization/center-biologics-evaluation-and-research-cber

28
https://www.fda.gov/about-fda/fda-organization/center-devices-and-radiological-health

29
https://www.fda.gov/medical-devices/how-study-and-market-your-device/breakthrough-devices-program

30
https://www.fda.gov/drugs/pharmaceutical-quality-resources/current-good-manufacturing-practice-cgmp-regulations

31
https://www.accessdata.fda.gov/scripts/cdrh/cfdocs/cfcfr/cfrsearch.cfm?fr=820.30

32
https://www.iso.org/standard/59752.html

33
https://www.accessdata.fda.gov/cdrh_docs/reviews/K071128.pdf

34
https://www.akoyabio.com/

35
https://www.akoyabio.com/wp-content/uploads/2020/11/20201026-Michael-McLane-SITC-A-novel-HnE-like-staining-method.pdf

36
https://indicalab.com/

37
https://visiopharm.com/

38
https://qupath.github.io/

39
https://akoyabio.github.io/phenoptr/

40
https://akoyabio.github.io/phenoptrReports/

41
https://github.com/

42
https://www.cms.gov/medicare-coverage-database/view/article.aspx?articleid=52,986&ver=183&/

43
https://www.ama-assn.org/amaone/cpt-current-procedural-terminology

44
https://www.r-project.org/

45
https://www.mathworks.com/products/matlab.html

46
https://www.python.org/

47
https://research.google/pubs/pub49953/

48
https://www.fda.gov/medical-devices/premarket-approval-pma/pma-application-contents

49
https://www.fda.gov/medical-devices/premarket-submissions-selecting-and-preparing-correct-submission/premarket-notification-510k

50
https://www.cms.gov/

51
https://www.fda.gov/regulatory-information/search-fda-guidance-documents/technical-performance-assessment-digital-pathology-whole-slide-imaging-devices

52
https://www.fda.gov/medical-devices/software-medical-device-samd/good-machine-learning-practice-medical-device-development-guiding-principles

53
https://www.dicomstandard.org/

54
https://www.color.org/index.xalter

55
https://www.image-net.org/challenges/LSVRC/

56
https://tupac.grand-challenge.org/

57
https://camelyon17.grand-challenge.org/

58
https://acdc-lunghp.grand-challenge.org/

59
https://www.kpmp.org/

60
https://www.imdrf.org/

61
https://ec.europa.eu/info/law/law-topic/data-protection_en

62
https://www.fda.gov/medical-devices/digital-health-center-excellence

63
https://www.who.int/

64
https://www.nist.gov/

65
https://www.bipm.org/en/

66
https://www.abcam.com/primary-antibodies/tackling-the-reproducibility-crisis

67
https://www.nordiqc.org/

68
https://ukneqasiccish.org/

69
https://cpqa.ca/main/

70
https://rcpaqap.com.au/

71
http://www.iqnpath.org/

72
https://www.fda.gov/regulatory-information/search-fda-guidance-documents/expedited-programs-serious-conditions-drugs-and-biologics

73
https://www.fda.gov/medical-devices/in-vitro-diagnostics/laboratory-developed-tests

74
https://documents.cap.org/documents/2015-fda-ldt-workshop-cap.pdf

75
https://www.accessdata.fda.gov/scripts/cdrh/cfdocs/cfcfr/CFRSearch.cfm?CFRPart=864

76
https://www.agilent.com/en/dako-products

77
https://diagnostics.roche.com/global/en/about/roche-tissue-diagnostics.html

78
https://www.danaher.com/our-businesses/diagnostics/leica-biosystems

79
https://www.dana-farber.org/research/departments-centers-and-labs/integrative-research-centers/center-for-cancer-genomics/profile/

80
https://www.cms.gov/medicare-coverage-database/view/lcd.aspx?lcdid=36675&ver=15&bc=0

81
https://tissuecypher.com

82
https://www.ema.europa.eu/en/human-regulatory/overview/medical-devices

83
https://eur-lex.europa.eu/legal-content/EN/ALL/?uri=CELEX%3A31998L0079

84
https://eur-lex.europa.eu/legal-content/EN/TXT/?uri=CELEX%3A32017R0746

85
https://www.congress.gov/bill/117th-congress/house-bill/4128

86
https://www.congress.gov/bill/117th-congress/senate-bill/1666

87
https://www.cancerresearch.org/fda-approval-timeline-of-active-immunotherapies

88
https://www.breastcancer.org/screening-testing/oncotype-dx

89
https://www.cancertherapyadvisor.com/home/news/conference-coverage/esmo-2022/breast-cancer-tnbc-ici-tumor-lymphocyte-level-higher-treatment-risk/

90
https://investors.akoyabio.com/news-releases/news-release-details/akoya-biosciences-partner-acrivon-therapeutics-clinical

91
https://acrivon.com/news-press/akoya-biosciences-to-partner-with-acrivon-therapeutics-for-the-clinical-development-of-acrivons-proprietary-oncosignature-test-into-a-companion-diagnostic/

92
https://www.decibio.com/market-reports/spatial-biology-market
